# Structural and Evolutionary Adaptation of NOD-Like Receptors in Birds

**DOI:** 10.1155/2021/5546170

**Published:** 2021-04-29

**Authors:** Xueting Ma, Baohong Liu, Zhenxing Gong, Xinmao Yu, Jianping Cai

**Affiliations:** ^1^State Key Laboratory of Veterinary Etiological Biology, Key Laboratory of Veterinary Parasitology of Gansu Province, Lanzhou Veterinary Research Institute, Chinese Academy of Agricultural Sciences, Xujiaping 1, Lanzhou, Gansu Province 730046, China; ^2^Jiangsu Co-Innovation Center for Prevention and Control of Animal Infectious Diseases and Zoonoses, Yangzhou, Jiangsu Province 225009, China

## Abstract

NOD-like receptors (NLRs) are intracellular sensors of the innate immune system that recognize intracellular pathogen-associated molecular patterns (PAMPs) and danger-associated molecular patterns (DAMPs). Little information exists regarding the incidence of positive selection in the evolution of NLRs of birds or the structural differences between bird and mammal NLRs. Evidence of positive selection was identified in four avian NLRs (NOD1, NLRC3, NLRC5, and NLRP3) using the maximum likelihood approach. These NLRs are under different selection pressures which is indicative of different evolution patterns. Analysis of these NLRs showed a lower percentage of codons under positive selection in the LRR domain than seen in the studies of Toll-like receptors (TLRs), suggesting that the LRR domain evolves differently between NLRs and TLRs. Modeling of human, chicken, mammalian, and avian ancestral NLRs revealed the existence of variable evolution patterns in protein structure that may be adaptively driven.

## 1. Introduction

Nod-like receptors (NLRs) are pivotal sensor proteins of the innate immune system with diverse functions. They detect pathogen-associated molecular patterns (PAMPS) of invading microbes and danger-associated molecular patterns (DAMPs), thus initiating an innate immune response.

Structurally, members of the NLR family share a similar tripartite domain organization: a variable N-terminal domain, a central nucleotide-binding and oligomerization domain (NACHT), and a C-terminal domain [1].

NLRs are grouped into subfamilies based on their specific N-terminal protein-protein interaction domain: the caspase recruitment domain (CARD), the pyrin domain (PYD), the baculovirus inhibitor of apoptosis protein repeat (BIR) domain, or an acidic transactivation domain [1, 2]. These N-terminal domains are involved in recruiting downstream effector molecules [2] and signal transduction. Moreover, the N-terminal region of an additional subfamily, NLRX1, contains a mitochondrial targeting sequence that shares no homology to any other protein [3, 4]. The central NACHT domains are similar to the STAND (signal-transduction ATPases with numerous domains) subclade of the AAA+ ATPases superfamily [5–7]. The central NACHT domain is required for protein oligomerization [1, 8].

NLRs detect PAMPS via a C-terminal leucine-rich repeats (LRR), which is a 20-29 residue repeated sequence with conserved and characteristically spaced hydrophobic residues [9, 10]. Recently, about 23 NLRs and NLR-like proteins have been reported in humans [11]. NLRs, including NLRP1, NLRP3, NLRP6, NLRP7, NLRC4, NLRP12, and NAIP, have been observed to activate the assembly of inflammasomes and mediate caspase-1 activation, leading to inflammation and cell pyroptosis [12, 13]. Other NLRs, such as NOD1, NOD2, NLRC3, NLRC5, NLRP10, NLRX1, and CIITA, have been reported to induce the activation of nuclear factor-kB (NF-*κ*B) and the mitogen activated protein kinase (MAPK) signaling pathway, or act as transcriptional regulators [12, 13].

Most studies on NLRs have focused on NLRs of mammals and fish. Recent studies on NLRs of early diverging organisms have suggested adaptive evolution in NLRs. In *Hydra*, which have large and complex NLR repertoires, NLRs recruit downstream adaptor molecules after activation. These NLRs, with the associated adaptor proteins, may induce apoptosis or activate putative NF-*κ*B/JNK transcription factors, thus regulating downstream cell responses and the expression of antimicrobial peptide [14]. It has been suggested that NLRs are ancient genes with putative cytoplasmic defense functions in basal metazoans and a common metazoan ancestor [14]. Gene expansion and domain gain, loss, and shuffling have occurred in the NLRs of *Hydra* and many other animals, indicating that NLRs have evolved in a species-specific manner to adapt to various ecological niches [14, 15].

In recent years, several important structures and functions of NLRs have been discovered in mammals. However, due to the different environmental conditions and ecological niches occupied by mammals and birds, conceivably their NLRs have evolved differently, generating unique structural and functional properties. Although evolution of the NOD subfamily of NLRs is known to be conserved in mammals, NOD2 is missing in chickens. In mammals, NOD2 senses specific bacterial muramyl dipeptides (MDP). In contrast to NOD2 in mammals, MDP are recognized by NLRP3 in chickens, suggesting that chicken NLRP3 replaces the role of mammalian NOD2 [16]. In mammals, NLRC5 is an important regulator of the MHC class-I antigen presentation pathway [17, 18]; however, one report showed no direct relationship between NLRC5 knockdown and MHC-I expression in chickens [19]. Moreover, the NLRP3 PYD domain, which is missing in *Xenopus* and zebrafish, has been identified in chickens [20]. Collectively, these observations suggest a pattern of molecular evolution of NLRs in vertebrates that is different from birds. Thus, the innate immune systems in all organisms, from *Hydra* and corals, to fish, birds, and mammals, have evolved in response to environmental conditions.

The aim of this work was, therefore, to identify evidence of positive selection in avian NLRs, to examine the structural and functional evolution of NLRs in birds, and to further elaborate the structural and functional diversity of NLRs between mammals and birds. Using Maximum Likelihood method, evidences of long-term selective pressure in the NLR genes have been found. To better understand how the CARD-binding properties of NLRs structurally and functionally diversified, ancestral NLR proteins were reconstructed and the structures of ancestral CARDs compared. Data from this study may provide more evidence for the role of positive selection in the evolution of NLRs.

## 2. Results

**Figure 1:**
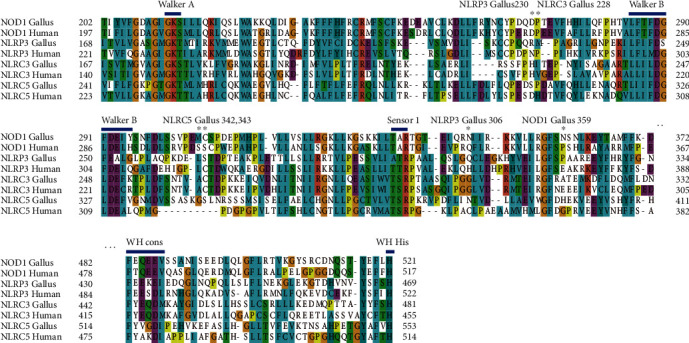
Adaptive substitutions in avian NLRs NACHT domain. We have aligned the NACHT domain of NLRs (human and Gallus shown). Adaptive amino acid substitutions and important motif of NACHT are indicated along the top of the alignment. Adaptive amino acid substitutions in NACHT are represented with star (^∗^); motifs involved in ATP binding and hydrolysis of NACHT are indicated along the top of the alignment.

Our study identified some positive selection sites in the pathogen recognition domains of NLRs that were examined. To evaluate the functional significance of the inferred positively selected sites, the location for each putatively positive site has been summarized in supplementary materials (Table 1). Our results provide evidence of positive selection in the NACHT domain of the avian NOD1, NLRC3, NLRP3, and NLRC5 (supplementary materials Table 1 and Figure 1). The NACHT domain plays a crucial role in the NLRs. It contains several characteristic motifs, namely, Walker A, Walker B, Sensor 1, and WH motif [21].

It has been reported that the sequence variations in the NACHT domain in human NLRs, especially in the vicinity of conserved regions or motifs, may influence the cycle of nucleotide-binding, -hydrolysis, -release, and/or conformational changes induced by NTP-hydrolysis, thus leading to inflammatory disorders [22, 23]. To investigate the effects of positive selection on the NACHT domain in four molecules, the positions of conserved motifs and residues under selective pressure were analyzed (Figure 1). None of observed positively selected codons were located within or close to the conserved motifs required for ATPase activity, activation, and oligomerization. As shown in the multiple sequence alignment between the *Gallus* and human sequences (Figure 1), motifs involved in NACHT functions are conserved among NLR proteins.

### 2.1. NLRC5

**Figure 2:**
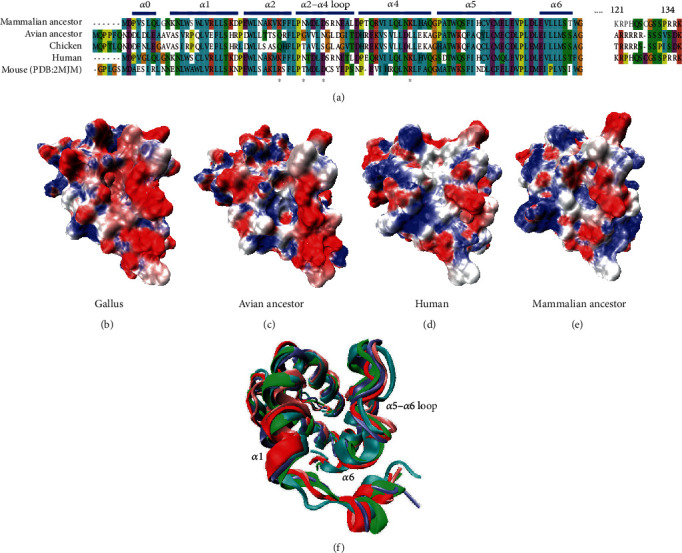
Sequence and primary functional differences in NLRC5 CARD are established (human, chicken, mouse, mammalian ancestor, and avian ancestor). (a) Sequence alignments of NLRC5 CARD domain. Adaptive amino acid substitutions in birds are represented with star (^∗^). Secondary structures of protein are shown along the top of the alignment. NLS (amino acids 121 to 134 in human) are shown. (b–e) The atypical CARD of NLRC5 consists of five *α*-helices (*α*1, *α*2, *α*4, *α*5, and *α*6) that are packed around a hydrophobic core. The CARD shape and electrostatic potential for human, chicken, mouse, mammalian ancestor, and avian ancestor are plotted. The surfaces are color-coded according to electrostatic surface potential: red, −10 kT; white, 0 kT; and blue, +10 kT. (f) Structural alignment of human, chicken, mammalian ancestor, and avian ancestor NLRC5 atypical CARD.

The quantity and distribution of positively selected codons varied among NLRs. The highest accumulation of positively selected sites occurred in NLRC5. Many sites under positive selection were located in N-terminal and C-terminal domains of the avian NLRC5. Remarkably, NLRC5 lacked the canonical N-terminal domain present in other defined domains of NLRs [24]. The N-terminal domain of NLRC5 is considered an atypical CARD. NLRC5 has a bipartite nuclear localization signal (NLS) which is required for nuclear localization of NLRC5 [17]. The NLS, the transport signal for nuclear protein import [25], is highly conserved in mammals and contains critical residues, i.e., Lys121, Arg122, Arg132, Arg133, and Lys134 in humans [26, 27]. Surprisingly, our sequence alignment indicates that the NLS (amino acids 121 to 134) was mostly absent in the *Gallus* and the avian ancestor NLRC5 sequences (Figure 2).

**Figure 3:**
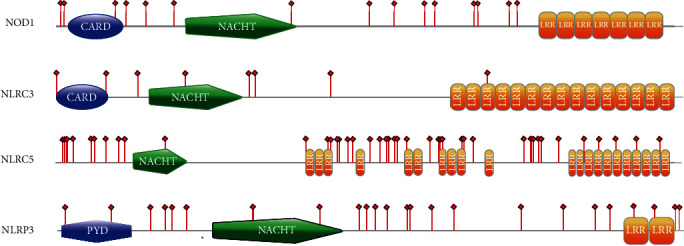
Location of positively selected sites of four NLR molecules in birds. Protein architectures were generated with PROSITE MyDomains image creator tool (http://www.expasy.org/tools/mydomains/). Red bars indicate adaptive amino acid substitutions. Colored areas represent different conserved domains (blue, CARD or PYD domain; green, NACHT domain; orange, LRR domain).

The highest accumulation of positively selected sites was observed in the LRR region of NLRC5 (Figure 3 and supplementary materials Table 1). NLRC5 contains a large LRR region that is different from other NLRs, with varying number of LRRs [26, 28]. To gain more insight into the functional significance of the putatively selected sites in LRRs, LRRSearch (http://www.lrrsearch.com) [29] was used to analyze the number and the location of the LRR of the chicken NLRC5. In general, the LRR domain contains 20–29 amino acid residues. It has a highly conserved segment (HCS) that consists of a consensus sequence and a variable segment (VS), which is located before the HCS of the next LRR. The HCS contains the consensus sequence LxxLxLxxN/CxL [10, 30, 31]. Of the 39 codons identified under positive selection in the LRRs of NLRC5, only 5 were localized in the HCS.

### 2.2. NOD1

**Figure 4:**
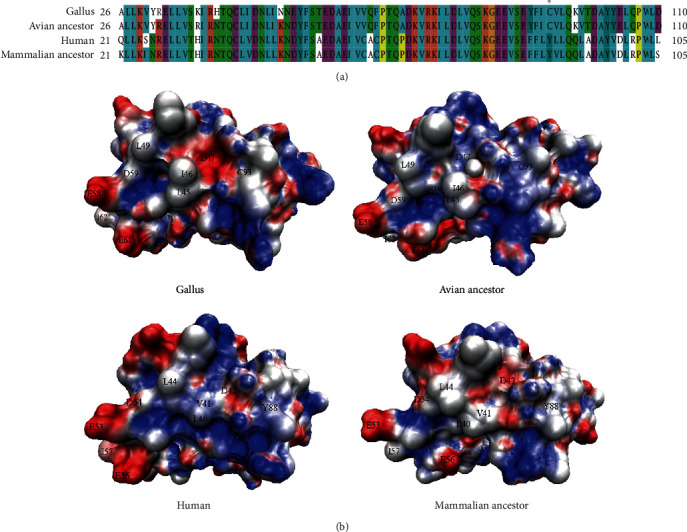
Sequence and primary functional differences in NOD1 CARD. (a) Alignment of NOD1 CARD domain sequences. Star (^∗^) indicates adaptive amino acid substitutions of NOD1 CARD in birds. (b) The NOD1 CARD shape and electrostatic potential for human, chicken, mouse, mammalian ancestor, and avian ancestor are plotted; the surfaces are color-coded according to electrostatic surface potential: red, −10 kT; white, 0 kT; and blue, +10 kT. Residues reported to be important for NOD1/RIP2 interaction, and NF-*κ*B activation in human is labeled.

The percentage of NOD1 sites under positive selection that were located in the known functional domains was low. In this study, only two positively selected sites (93 and 359) have been found within the functional domains. Amino acids 93 and 359 were located within the CARD domain and NACHT domain of avian NOD1, respectively. In the avian NOD1, Cys93 was replaced by Tyr, His, Arg, Leu, Phe, and Ser. Because the crystal structure of the human NOD1 CARD has been resolved and residues involved in the downstream signaling and interaction with RIP2 are recognized [32], we aligned the sequences of NOD1 CARD domain and plotted the CARD domain structure and electrostatic potential for human and *Gallus* NOD1 as well as for reconstructed ancestral NOD1 proteins (Figure 4). The results obtained henceforth suggested that the positively selected site 93 is not a key residue in the NOD1-RIP2 interaction. Since a similar degree of positive selection in interacting molecules is expected, we analyzed the positively selected codons in RIP2 (supplementary materials Table 1). Amino acids 324, 371, and 412 were identified to be under positive selection but were not located within the CARD domain of RIP2.

### 2.3. NLRC3 and NLRP3

It has been reported that NLRC3, together with NOD1, originated from a gene duplication event that occurred before the divergence of birds and mammals [33]. A minimum number of adaptive substitutions were present within the avian NLRC3s (supplementary materials Table 1 and Figure 3), showing a stabilized selection pattern of evolution. Although the functions of many substitutions are unknown, they would support the hypothesis that species-specific adaptations occur because of different environmental conditions and pathogens. NLRP3 participates in the assembly of inflammasome complexes. Mutations in NLRP3 are associated with many inflammatory diseases in humans [34]. Our analysis of avian NLRP3s detected many amino acid sites that are under positive selection (supplementary materials Table 1 and Figure 3).

## 3. Discussion

Evidence of positive selection in the bird NLRs is reported in this study with variable quantity and distribution of positively selected residues.

### 3.1. NOD1

NOD1 is a crucial molecule in innate immunity. It activates the NF-*κ*B pathway by recruiting the protein receptor-interacting protein 2 (RIP2) through the interaction of CARDs. Two positively selected codons have been found in the functional domain of the *Gallus* NOD1, amino acids 93 and 359. Interactions between NOD1 and RIP2 mainly depend on electrostatic interactions in the CARD domain [32]. The CARD domain, as an important effector domain, belongs to the death domain superfamily. Members of the death domain superfamily always promote homotypic and/or heterotypic interactions with other effector domain containing proteins.

In NOD1, the CARD domain is indispensable for downstream signaling, involved in many different cellular processes, such as apoptosis and inflammation. Because of functional constraints, residues with critical functions may be under slow evolutionary rate [35]. It has been reported that the core residues which constitute the polar surface and the hydrophobic core of CARD are conserved [36]. As expected, the CARD domain of NOD1 had a lower percentage of positively selected codons than other NLRs, and it showed greater selective constraints.

To have further insight of the functional evolution of the NOD1 CARD and to understand if the positively selected residue (residue 93, located in *α*5) has the potential to influence the function of the CARD, we predicted the ancestral amino acid state of the CARD for mammals and birds and analyzed its 3D structure. The CARD domain of human NOD1 consists of six antiparallel *α*-helices packed around a hydrophobic core with residues E53, D54, and E56 of *α*3 involved in the interaction with RIP2 [32]. It has been reported that the surface shape of CARD and electrostatic interaction of oppositely charged residues plays a significant role in CARD-CARD interactions [32, 36, 37]. Mutations in L44, I57, and V41 greatly reduced activation of NOD1 signaling which demonstrated the significance of hydrophobic residues [32].

Our alignment results (Figure 4) showed that the ancestral CARD of mammals and avian species are conserved. Not only are the residues buried in the hydrophobic core and involved in interactions of the avian ancestor relatively well conserved but the shape and electrostatic potential of the binding area of the avian ancestor are very similar to human and chicken NOD1 (Figure 4). In human and mammalian ancestor, the NOD1 CARD domain residue 93 was replaced by Tyr, but the electrostatic distribution in this region was not altered (Figure 4). We examined the location of amino acid 93 and found that it was buried, leaving the electrostatic distribution on the surface of CARD unchanged. Similarly, analysis of avian RIP2 showed that the residues (R486, R525, and R530) of the RIP2 CARD domain involved in the interaction with NOD1 CARD were not under positive selection (supplementary materials Table 1), and these residues were conserved between avian and human (supplementary materials S1). This is consistent with its important biological function.

### 3.2. NLRC5

The N-terminal domain of NLRC5, which contains repeated *α*-helices and is strikingly distinct from other CARDs, is referred to as an atypical CARD. To investigate the structural differences of atypical CARDs, we compared the human, chicken, mammalian, and avian ancestral structures using homology modeling on an atypical mouse CARD, whose conformation is known [38]. Atypical CARD of NLRC5 consists of five *α*-helices that are packed around a hydrophobic core, with the difference to other CARDs (such as NOD1 CARD) being the absence of *α*3, which was replaced with an *α*2-*α*4 loop. Interestingly, other studies of NOD1 CARD have suggested conserved *α*2 and *α*3 domains which form a putative interaction surface with the CARD of binding partner [36]. In our study, no positive selection is identified in *α*2 and *α*3 of CARD domains in NOD1 (positive selection 93 codon is in CARD *α*5 of NOD1). NLRC5, however, also contacts with the binding partner by CARD-CARD interaction and contains four positively selected sites that fall in the *α*2, *α*4, and *α*2-*α*4 loop of atypical CARD domain (supplementary materials Table 1 and Figure 2).

NOD1 interacts with its binding partner RIP2 by CARD-CARD interaction mainly through complementary surface shape and charge [38]. The hydrophobic interactions, however, make contributions to CARD-CARD interactions between NLRC5 and its partner RIG-I [38]. Moreover, the atypical CARD of NLRC5 interacts with the binding partner's CARD by its hydrophobic surface which is composed of *α*1, *α*6, and the *α*5–*α*6 loop but not the *α*2 and *α*2-*α*4 loop [38]. Our alignment (Figure 2) showed that, except residues belonging to *α*0, *α*2, *α*4, and the *α*2-*α*4 loop, most residues of the atypical CARD were conserved in chicken, human, mouse, and avian and mammalian ancestors. For avian, positively selected sites mainly fall in the *α*2, *α*4, and *α*2-*α*4 loop of NLRC5 (Figure 2); the *α*1, *α*6, and the *α*5–*α*6 loop of NLRC5 that encode the RIG-I-binding region were more conserved than the *α*2 and the *α*2-*α*4 loop. A possible explanation may be that the residues in the RIG-I-binding region are directly involved in downstream signal transduction. The 3D structures of atypical CARDs were generally similar in the human, chicken, mouse, mammalian, and avian ancestors (Figure 2). But the electrostatic potentials of the pocket formed by *α*1, *α*6, and the *α*5–*α*6 loop in the chicken and the avian ancestor are different from both the human and mammalian ancestral atypical CARDs which indicates a different evolution pattern of NLRC5 between birds and mammals.

Our additional alignment studies showed that the NLS (amino acids 121 to 134) was missing in the CARDs of bird NLRC5s (Figure 2). Sequence analysis of the chicken NLRC5 performed by cNLS Mapper [39] revealed a putative monopartite NLS at amino acids 1012-1025, which do not belong to CARD domain. In the avian ancestor, the NLS has been found absent using the cNLS Mapper prediction algorithm. The NLS is the transport signal for nuclear protein import [25] and is well conserved in mammals.

NLRC5 is an IFN-*γ*–inducible nuclear protein recognized as an important molecule for the MHC class-I antigen presentation pathway in humans [17, 18, 24]. Within the NLS sequence context, NLRC5 associates with promoters of MHC class-I genes and activates them, inducing expression of MHC class-I [17, 18]. Mutations within the NLS may prevent nuclear localization [17] and thereby reduce induction of MHC class-I expression [27]. It has, however, been reported that in chicken macrophages, there is no difference in MHC class-I gene expression between NLRC5 knockdown cells and controls, suggesting that NLRC5 may be a dispensable regulator of MHC class-I in chicken macrophages [19]. In zebrafish, overexpression of NLRC5 induces the expression of MHC class-II genes but not MHC class-I genes [40]. These findings indicate the functional divergence of NLRC5 between mammals, birds, and fishes.

### 3.3. NLRC3

**Figure 5:**
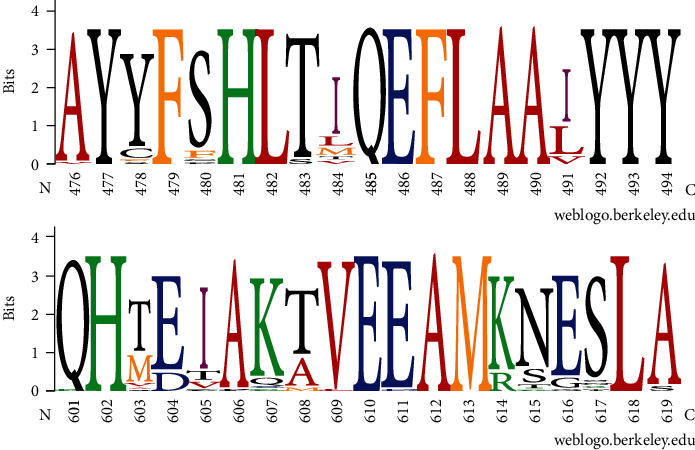
WebLogo of the putative TRAF2-binding sites constructed from alignment of 41 birds NLRC3 sequences. The residue numbering corresponds to the residues from chicken NLRC3. TRAF-binding motif mainly contains four residues with (P/S/A/T)-X-(Q/E)-E in the NACHT domain; “P/S/A/T” represents Pro, Ser, Ala, or Thr; “X” indicates any amino acid; “Q/E” represents Gln or Glu. Major TRAF2-binding motifs in birds NLRC3 are at the region of 483-486 and 608-611. The letter size is proportional to the degree of amino acid conservation. The weblogo was made using the web-based application WebLogo (http://weblogo.berkeley.edu).

A minimal number of adaptive substitutions have been found within NLRC3 (supplementary materials Table 1 and Figure 3), suggesting a stabilized selection pattern in evolution. It is difficult to speculate about the impact of functional effects caused by substitutions in NLRC3 without an available crystal structure. Recent studies have suggested that NLRC3 functions to regulate the strength and time of the inflammatory response, suppress the activation of various innate immune signaling cascades, and prevent excessive immune responses [41, 42]. NLRC3 can regulate the STING signaling pathway and the TRAF6 signaling pathway. Also, NLRC3 negatively modulates STING activation via the response to cytosolic DNA, cyclic di-GMP, and DNA viruses [41]. Moreover, NLRC3 negatively regulates the activation of NF-*κ*B through interaction with TRAF6 [42]. It has been demonstrated that an interaction between the NACHT domain of NLRC3 and STING is mediated by the LRR domain [41]. Thus, NLRC3 may decrease STING dependent innate immune activation [41]. The NACHT domain of NLRC3 also affects the NF-*κ*B pathway by binding with TRAF6 [42]. NLRC3 binds to TRAF6 via the TRAF-binding motif in the NACHT domain [42]. NLRC3 has two TRAF-binding motifs that are conserved among various species [42]. Our analysis found conserved TRAF2-binding sites in birds (Figure 5). NLRC3 has been reported to be an inhibitor of the PI3K-AKT-mTOR pathways [43]. Moreover, an NLRC3-like protein of zebrafish has a negative regulatory function on macrophage activation and inflammation. It has been reported that both the PYD and the NACHT domains are essential for function of the NLRC3-like protein in zebrafish, and loss of function mutations in the zebrafish NLRC3-like protein may result in systemic inflammation [44]. This provides support for cross-species functionality of NLRC3 [43]. Hence, it is possible that mutations in NLRC3 are likely to influence inflammatory signaling pathways and even induce inflammatory disorders. These studies, collectively, provide reasonable evidence that NLRC3 evolved under strong stabilizing selection.

### 3.4. NLRP3

NLRP3 is a member of the NLR family and belongs to the NLRP subclass characterized by the presence of a PYD. Human NLRP3 is activated in response to a broad range of stimuli including bacteria, fungi, yeast, viruses, parasites, pore-forming toxins, crystals, TLR ligands, bacterial RNA, and DAMPs, such as ATP and hyaluronan [45–48]. NLRP3 participates in the assembly of inflammasome complexes, which in most cases, help the host eliminate invading pathogens. Aberrant accumulation of inflammasome signals may result in disease in humans. Mutations in and around the NACHT of NLRP3 cause three auto-inflammatory diseases: FCAS (familial cold auto-inflammatory syndrome), MWS (Muckle–Wells Syndrome), and CINCA/NOMID (chronic infantile neurological cutaneous and articular syndrome/neonatal onset multisystemic auto-inflammatory disease) [34]. Several amino acids showed evidence of positive selection that were detected in or around the NACHT domain of NLRP3 in the chicken and avian ancestor sequences we examined (Figures 1 and 3).

A relatively recent study has demonstrated that the mutations may affect the stabilization of NLRP3 by increasing its half-life [49], showing that the NLRP3 protein, stable at lower temperatures, was degraded at 37°C [49]. In avian species, high metabolic rates may lead to high body temperatures—the mean body temperatures for all birds has been reported to be 38.54°C (±0.96) for resting birds, 41.02°C (±1.29) for birds in the active phase, and 43.85°C (±0.94) for highly active birds [50]. These findings suggest that positive selection found in or around the NACHT domain of bird NLRP3 may be associated with adaptation to a higher body temperature in birds. In the chicken, NOD2 was replaced with NLRP3 as another potential PRR to sense MDP [16]. Recent studies have reported that the NLRP3 gene is variable between mammalian and avian species [51]. The PYD domain of NLRP3 was not identified in *Xenopus* or the zebrafish [20]. Another inflammation-related molecule, IL-1*β*, has also been identified to be highly variable between mammalian and avian species, suggesting potentially different mechanisms in the host inflammatory responses between these classes [51].

### 3.5. LRR Domains of NLRs

Previous research has shown a high number of positively selected codons located in LRR domain of TLRs [52]. Our results, however, indicate a different level of positive selection acting in LRR domain of NLRs. NLRC5, NOD1, NLRC3, and NLRP3 showed lower percentage of codons under positive selection in the LRR domain than TLRs. The TLRs are transmembrane proteins which recognize PAMPs through the extracellular domain of LRRs. The NLRs are intracellular, cytoplasmic sensors. Recent studies have emphasized that interactions between NLRs and PAMPs may be indirect and involve intermediates of host cells, which like R proteins indirectly sense PAMPs in plants [1, 53, 54]. This may explain the different patterns of evolution observed for LRRs in TLRs and NLRs. NLRC5 contains a large LRR region that is different from other NLRs, with the exact number of LRRs being uncertain [26, 28]. LRRs of the human NLRC5 are involved in the interaction with IKK*α* and IKK*β*, blocking phosphorylation and degradation of the inhibitory I*κ*B proteins, thus, inhibiting NF-*κ*B activation [55]. In addition, the LRR region of human NLRC5 is responsible for nuclear export of NLRC5, as well as transcriptional activation [56]. Of the 39 codons identified under positive selection in the LRRs of the chicken and avian ancestral NLRC5, only 5 amino acids were localized in the HCS. Although the number of LRRs in NLRC5 is still controversial, the C-terminal LRR repeat of human NLRC5 has been reported to be of 36 amino acid residues in length [26] while others have suggested that the C-terminal capping motifs may serve to protect the protein [57]. Interestingly, recent studies have also described an NES (nuclear export signal) in the C-terminal region of LRRs that mediates nuclear export in humans [17].

It is reasonable to predict conserved evolution for this region. Our analyses mapped one codon under positive selection (residue 1800), which falls in the region closest to the C-terminal LRRs. Because of the large size of the LRR region and unusual structure of NLRC5, it has been speculated that NLRC5 might sense different stimuli, compared to other NLRs [28]. Some of the positively selected amino acid sites in LRRs may, thus, be involved in the recognition of these diverse stimuli. The detailed molecular basis of the role of NLRC5 in host defense and immune signaling, however, is largely still unknown.

## 4. Conclusion

The Nod-like receptors of the innate immunity represent the first line of defense against the pathogens. These are involved in pathogen recognition and, thus, need to evolve rapidly in a dynamic arms race with pathogens. Evidence of positive selection of NLRs in birds has been observed. Adaptive selection in avian NLRs was different than either avian TLRs or mammalian NLRs. NLRs have shown adaptive divergence in structure and function throughout the avian evolution. This work provides a basic understanding of structural and functional evolution in avian NLRs. By looking for structural differences between human, chicken, mammalian ancestral, and avian ancestral NLRs, we propose that the different environmental conditions encountered by mammals and birds might have induced structural and functional differentiation among members of the NLR family. Different NLRs with varied functions might have experienced unique pressures which might have induced the evolutionary change.

## 5. Materials and Methods

### 5.1. Data Collection

The coding regions of three chicken (Lingnanhuang: LNH) NOD-like-receptor genes, NOD1, NLRC3, and NLRP3, were obtained by polymerase chain reaction (PCR) amplification using gene-specific primers (primer details in supplementary materials S2). Chicken NLRC5 and all avian NLR sequences (accession number displayed in supplementary materials S3) were collected from NCBI (http://www.ncbi.nlm.nih.gov). NOD-like-receptor amino acid sequences of chicken, avian ancestors, and mammalian ancestors are displayed in supplementary materials S2.

### 5.2. Sequence Alignment

Sequence alignments were produced using PROBCONS version 1.12 [58]. The alignments used for phylogenetic analyses were processed using Gblocks v0.91b [59] to detect and filter potentially unreliable and misaligned regions. To detect positive selection in individual codons of the NLR sequences, a phylogenetic tree was reconstructed by MrBayesv3.2.2 (http://mrbayes.csit.fsu.edu/) [60]. All analyses were run with 2000,000 generation in Mrbayes. Convergence was considered to have been achieved with split frequency values of <0.01. If the split frequency did not drop below 0.01, the analysis continued with additional 2000,000 generations. The first 25% of the topologies were discarded as burn-in. Sequence analysis of the chicken NLRC5 was performed by cNLS Mapper (http://nls-mapper.iab.keio.ac.jp/cgi-bin/NLS_Mapper_form.cgi) [39].

### 5.3. Tests of Selection

All sequences for testing positive selections have been displayed in supplementary materials S3. To test positive selections at NLRs, the ratios of nonsynonymous (dN) to synonymous (dS) substitutions per site were compared in a maximum likelihood (ML) framework. A ratio of dN/dS > 1 is interpreted as evidence of positive selection. In order to improve the accuracy of the positive selection results, two ML frameworks were selected, the codeml program of PAML [61] and the HyPhy package of the Data Monkey Web Server (http://www.datamonkey.org) [62]. For codeml, two alternative models (M7 and M8) were chosen and compared with twice the difference of log-likelihood value (2ΔlnL) with 2 degrees of freedom to investigate whether sites were under positive selection in each NLRs. For codeml, the Bayes Empirical Bayes (BEB) posterior probability method was calculated in conjunction with site models to identify individual codons as adaptive [63]. In HyPhy, three distinct models, SLAC, FEL, and REL, were conducted; the level of statistical significance was set at a *p* value =0.1 for SLAC and FEL and Bayes Factor = 50 for REL analysis, sites with a *p* value <0.1 for SLAC and FEL, and Bayes Factor > 50 for REL was accepted to identify candidates for selection.

Protein-coding substitutions identified as more than or equal to two ML methods were considered adaptive. In order to identify robust sites under positive selection, only sites with evidence of selection in at least two of the ML methods were considered. In addition, the PAL2NAL program [64] was used to convert the amino acid alignment into a codon-based DNA alignment for PAML codeml test.

### 5.4. Ancestral Sequence Reconstruction

Ancestral protein sequences were reconstructed using MEGA 6 [65] with the maximum likelihood algorithm. All nucleotide sequences were converted to corresponding protein sequences were aligned with PROBCONS. For each alignment, the best fitting nucleotide substitution model for each codon position was evaluated using the AIC criterion in MEGA with “find best DNA/Protein models.” An evolutionary tree was reconstructed with the ML method. For each internal node of the tree, MEGA exported a file including information of most probable ancestral sequences, and program ExtAncSeqMEGA [66] could then extract the ancestral DNA and protein sequences from the file. The accuracy scores of ancestral DNA and protein sequences were estimated. All ancestral sequences have been displayed in supplementary materials S2.

### 5.5. Homology Modeling and Structural Analysis

To obtain a more precise idea of the functional evolution of avian NLRs, structural homology models are built with MODELLER v9.5 [67]. Then, the structures were processed using the PDB2PQR server (http://nbcr-222.ucsd.edu/pdb2pqr_2.0.0/) [68] to add hydrogen atoms and force field parameters. Electrostatic surface potentials were estimated with APBS web solver (Adaptive Poisson Boltzmann Solver). The visualization of structures and electrostatic potential maps was generated with VMD1.9.1 [69].

## Data Availability

GenBank accession numbers of chicken (Lingnanhuang) NLR nucleotide sequences (generated in the course of the study) are as follows: MT385526, MT385527, and MT385528. The chicken sequences (third-party data) and ancestral sequences are deposited in Supplementary File S2. The accession number of avian nucleotide sequences analyzed in the article is deposited in Supplementary File S3.

## Additional Points

*Significance Statement.* Despite several studies on the evolution of TLRs in birds, a clear picture of the evolution of birds NLRs has not yet emerged. We have found evidence of positive selection of NLRs within the birds, reconstructed avian, and mammalian ancestral sequence of NLRs and compared the structural and functional differences of NLRs between mammals and avian. Different evolution patterns of the molecular binding region were specifically investigated in our study. Our analyses of avian NLR family provide more evidence for the role of positive selection in the evolution of NLRs.

## Supplementary Materials

Supplementary Materials S1: RIP2 alignment. S2: primers and chicken sequences and ancestral sequences. S3: avian sequences accession number. Supplementary Table 1: sites found to be under positive selection.

## References

[1] Chen G., Shaw M. H., Kim Y.-G., Nuñez G. (2009). NOD-like receptors: role in innate immunity and inflammatory disease. *Annual Review of Pathology: Mechanisms of Disease*.

[2] Ye Z., Ting J. P.-Y. (2008). NLR, the nucleotide-binding domain leucine-rich repeat containing gene family. *Current Opinion in Immunology*.

[3] Moore C. B., Bergstralh D. T., Duncan J. A. (2008). NLRX1 is a regulator of mitochondrial antiviral immunity. *Nature*.

[4] Arnoult D., Soares F., Tattoli I., Castanier C., Philpott D. J., Girardin S. E. (2009). An N-terminal addressing sequence targets NLRX1 to the mitochondrial matrix. *Journal of Cell Science*.

[5] Kufer T. A., Sansonetti P. J. (2011). NLR functions beyond pathogen recognition. *Nature Immunology*.

[6] Maekawa T., Kufer T. A., Schulze-Lefert P. (2011). NLR functions in plant and animal immune systems: so far and yet so close. *Nature Immunology*.

[7] Kadota Y., Shirasu K., Guerois R. (2010). NLR sensors meet at the SGT1-HSP90 crossroad. *Trends in Biochemical Sciences*.

[8] Monie T. P., Bryant C. E., Gay N. J. (2009). Activating immunity: lessons from the TLRs and NLRs. *Trends in Biochemical Sciences*.

[9] Rosenstiel P., Jacobs G., Till A., Schreiber S. (2008). NOD-like receptors: ancient sentinels of the innate immune system. *Cellular and Molecular Life Sciences*.

[10] Wei T., Gong J., Jamitzky F., Heckl W. M., Stark R. W., Rössle S. C. (2008). LRRML: a conformational database and an XML description of leucine-rich repeats (LRRs). *BMC Structural Biology*.

[11] Allen I. C. (2014). Non-inflammasome forming NLRs in inflammation and tumorigenesis. *Frontiers in Immunology*.

[12] Barbé F., Douglas T., Saleh M. (2014). Advances in Nod-like receptors (NLR) biology. *Cytokine & Growth Factor Reviews*.

[13] Zhu H., Cao X. (2017). NLR members in inflammation-associated carcinogenesis. *Cellular & Molecular Immunology*.

[14] Lange C., Hemmrich G., Klostermeier U. C. (2011). Defining the origins of the NOD-like receptor system at the base of animal evolution. *Molecular Biology and Evolution*.

[15] Hamada M., Shoguchi E., Shinzato C., Kawashima T., Miller D. J., Satoh N. (2013). The complex NOD-like receptor repertoire of the coral Acropora digitifera includes novel domain combinations. *Molecular Biology and Evolution*.

[16] Martinon F., Agostini L., Meylan E., Tschopp J. (2004). Identification of bacterial muramyl dipeptide as activator of the NALP3/cryopyrin inflammasome. *Current Biology*.

[17] Meissner T. B., Li A., Biswas A. (2010). NLR family member NLRC5 is a transcriptional regulator of MHC class I genes. *Proc. Natl. Acad. Sci. USA*.

[18] Yao Y., Wang Y., Chen F. (2012). NLRC5 regulates MHC class I antigen presentation in host defense against intracellular pathogens. *Cell Research*.

[19] Lian L., Ciraci C., Chang G., Hu J., Lamont S. J. (2012). NLRC5 knockdown in chicken macrophages alters response to LPS and poly (I:C) stimulation. *BMC Veterinary Research*.

[20] Laing K. J., Purcell M. K., Winton J. R., Hansen J. D. (2008). A genomic view of the NOD-like receptor family in teleost fish: identification of a novel NLR subfamily in zebrafish. *BMC Evolutionary Biology*.

[21] Proell M., Riedl S. J., Fritz J. H., Rojas A. M., Schwarzenbacher R. (2008). The Nod-like receptor (NLR) family: a tale of similarities and differences. *PLoS One*.

[22] Rosenstiel P., Till A., Schreiber S. (2007). NOD-like receptors and human diseases. *Microbes and Infection*.

[23] Albrecht M., Domingues F. S., Schreiber S., Lengauer T. (2003). Structural localization of disease-associated sequence variations in the NACHT and LRR domains of PYPAF1 and NOD2. *FEBS Letters*.

[24] Benko S., Magalhaes J. G., Philpott D. J., Girardin S. E. (2010). NLRC5 limits the activation of inflammatory pathways. *The Journal of Immunology*.

[25] Lange A., Mills R. E., Lange C. J., Stewart M., Devine S. E., Corbett A. H. (2007). Classical nuclear localization signals: definition, function, and interaction with importin *α*. *The Journal of Biological Chemistry*.

[26] Mótyán J. A., Bagossi P., Benkő S., Tőzsér J. (2013). A molecular model of the full-length human NOD-like receptor family CARD domain containing 5 (NLRC5) protein. *BMC Bioinformatics*.

[27] Meissner T. B., Li A., Liu Y.-J., Gagnon E., Kobayashi K. S. (2012). The nucleotide-binding domain of NLRC5 is critical for nuclear import and transactivation activity. *Biochemical and Biophysical Research Communications*.

[28] Neerincx A., Lautz K., Menning M. (2010). A role for the human nucleotide-binding domain, leucine-rich repeat-containing family member NLRC5 in antiviral responses. *The Journal of Biological Chemistry*.

[29] Bej A., Sahoo B. R., Swain B., Basu M., Jayasankar P., Samanta M. (2014). LRRsearch: an asynchronous server-based application for the prediction of leucine-rich repeat motifs and an integrative database of NOD-like receptors. *Computers in Biology and Medicine*.

[30] Kobe B., Kajava A. V. (2001). The leucine-rich repeat as a protein recognition motif. *Current Opinion in Structural Biology*.

[31] Matsushima N., Miyashita H., Mikami T., Kuroki Y. (2010). A nested leucine rich repeat (LRR) domain: the precursor of LRRs is a ten or eleven residue motif. *BMC Microbiology*.

[32] Manon F., Favier A., Núñez G., Simorre J.-P., Cusack S. (2007). Solution structure of NOD1 CARD and mutational analysis of its interaction with the CARD of downstream kinase RICK. *Journal of Molecular Biology*.

[33] Hughes A. L. (2006). Evolutionary relationships of vertebrate NACHT domain-containing proteins. *Immunogenetics*.

[34] Lamkanfi M., Kanneganti T.-D. (2010). Nlrp3: an immune sensor of cellular stress and infection. *The International Journal of Biochemistry & Cell Biology*.

[35] Knudsen B., Miyamoto M. M. (2001). A likelihood ratio test for evolutionary rate shifts and functional divergence among proteins. *Proceedings of the National Academy of Sciences of the United States of America*.

[36] Chou J. J., Matsuo H., Duan H., Wagner G. (1998). Solution structure of the RAIDD CARD and model for CARD/CARD interaction in caspase-2 and caspase-9 recruitment. *Cell*.

[37] Qin H., Srinivasula S. M., Wu G., Fernandes-Alnemri T., Alnemri E. S., Shi Y. (1999). Structural basis of procaspase-9 recruitment by the apoptotic protease- activating factor 1. *Nature*.

[38] Gutte P. G. M., Jurt S., Grütter M. G., Zerbe O. (2014). Unusual structural features revealed by the solution NMR structure of the NLRC5 caspase recruitment domain. *Biochemistry*.

[39] Kosugi S., Hasebe M., Tomita M., Yanagawa H. (2009). Systematic identification of cell cycle-dependent yeast nucleocytoplasmic shuttling proteins by prediction of composite motifs. *Proceedings of the National Academy of Sciences of the United States of America*.

[40] Wu X. M., Hu Y. W., Xue N. N. (2017). Role of zebrafish NLRC5 in antiviral response and transcriptional regulation of MHC related genes. *Developmental and Comparative Immunology*.

[41] Zhang L., Mo J., Swanson K. V. (2014). NLRC3, a member of the NLR family of proteins, is a negative regulator of innate immune signaling induced by the DNA sensor STING. *Immunity*.

[42] Schneider M., Zimmermann A. G., Roberts R. A. (2012). The innate immune sensor NLRC3 attenuates Toll-like receptor signaling via modification of the signaling adaptor TRAF6 and transcription factor NF-*κ*B. *Nature Immunology*.

[43] Karki R., Man S. M., Malireddi R. K. S. (2016). NLRC3 is an inhibitory sensor of PI3K-mTOR pathways in cancer. *Nature*.

[44] Shiau C. E., Monk K. R., Joo W., Talbot W. S. (2013). An anti-inflammatory NOD-like receptor is required for microglia development. *Cell Reports*.

[45] Kanneganti T.-D., Özören N., Body-Malapel M. (2006). Bacterial RNA and small antiviral compounds activate caspase-1 through cryopyrin/Nalp3. *Nature*.

[46] Mariathasan S., Weiss D. S., Newton K. (2006). Cryopyrin activates the inflammasome in response to toxins and ATP. *Nature*.

[47] Martinon F., Pétrilli V., Mayor A., Tardivel A., Tschopp J. (2006). Gout-associated uric acid crystals activate the NALP3 inflammasome. *Nature*.

[48] Willingham S. B., Bergstralh D. T., O'Connor W. (2007). Microbial pathogen-induced necrotic cell death mediated by the inflammasome components CIAS1/cryopyrin/NLRP3 and ASC. *Cell Host & Microbe*.

[49] Brydges S. D., Mueller J. L., McGeough M. D. (2009). Inflammasome-mediated disease animal models reveal roles for innate but not adaptive immunity. *Immunity*.

[50] Prinzinger R., Preßmar A., Schleucher E. (1991). Body temperature in birds. *Comparative Biochemistry and Physiology Part A: Physiology*.

[51] Ye J., Yu M., Zhang K. (2015). Tissue-specific expression pattern and histological distribution of NLRP3 in Chinese yellow chicken. *Veterinary Research Communications*.

[52] Areal H., Abrantes J., Esteves P. J. (2011). Signatures of positive selection in Toll-like receptor (TLR) genes in mammals. *BMC Evolutionary Biology*.

[53] Kawai T., Akira S. (2009). The roles of TLRs, RLRs and NLRs in pathogen recognition. *International Immunology*.

[54] Jones J. D., Dangl J. L. (2006). The plant immune system. *Nature*.

[55] Cui J., Zhu L., Xia X. (2010). NLRC5 negatively regulates the NF-*κ*B and type I interferon signaling pathways. *Cell*.

[56] Neerincx A., Rodriguez G. M., Steimle V., Kufer T. A. (2012). NLRC5 controls basal MHC class I gene expression in an MHC enhanceosome-dependent manner. *The Journal of Immunology*.

[57] Bella J., Hindle K. L., McEwan P. A., Lovell S. C. (2008). The leucine-rich repeat structure. *Cellular and Molecular Life Sciences*.

[58] Do C. B., Mahabhashyam M. S., Brudno M., Batzoglou S. (2005). ProbCons: probabilistic consistency-based multiple sequence alignment. *Genome Research*.

[59] Castresana J. (2000). Selection of conserved blocks from multiple alignments for their use in phylogenetic analysis. *Molecular Biology and Evolution*.

[60] Huelsenbeck J. P., Ronquist F., Nielsen R., Bollback J. P. (2001). Bayesian inference of phylogeny and its impact on evolutionary biology. *Science*.

[61] Yang Z. (2007). PAML 4: phylogenetic analysis by maximum likelihood. *Molecular Biology and Evolution*.

[62] Pond S. L., Frost S. D. (2005). Datamonkey: rapid detection of selective pressure on individual sites of codon alignments. *Bioinformatics*.

[63] Yang Z., Wong W. S., Nielsen R. (2005). Bayes empirical Bayes inference of amino acid sites under positive selection. *Molecular Biology and Evolution*.

[64] Suyama M., Torrents D., Bork P. (2006). PAL2NAL: robust conversion of protein sequence alignments into the corresponding codon alignments. *Nucleic Acids Research*.

[65] Tamura K., Stecher G., Peterson D., Filipski A., Kumar S. (2013). MEGA6: molecular evolutionary genetics analysis version 6.0. *Molecular Biology and Evolution*.

[66] Hall B. G. (2011). *Working with Various Computer Platforms Phylogenetic Trees Made Easy: A How-to Manual 5Edn*.

[67] Sali A., Blundell T. L. (1993). Comparative protein modelling by satisfaction of spatial restraints. *Journal of Molecular Biology*.

[68] Dolinsky T. J., Nielsen J. E., McCammon J. A., Baker N. A. (2004). PDB2PQR: an automated pipeline for the setup of Poisson–Boltzmann electrostatics calculations. *Nucleic Acids Research*.

[69] Humphrey W., Dalke A., Schulten K. (1996). VMD: visual molecular dynamics. *Journal of Molecular Graphics*.

