# Supplementary material for: Structural and Evolutionary Adaptation of NOD-Like Receptors in Birds
Source: Biomed Res Int. 2021 Apr 29;2021:5546170. doi: 10.1155/2021/5546170 (PMC8105094; doi:10.1155/2021/5546170)
Supplement: Supplementary Materials — S1: RIP2 alignment. S2: primers and chicken sequences and ancestral sequences. S3: avian sequences accession number. Supplementary Table 1: sites found to be under positive selection. [file 5546170.f1.zip › S1 RIP2 alignment.pdf]

>NP_003812.1_Homo_sapiens
MNG----------EAICSALPTIPYHKLADLRYLSRGASGTVSSARHADWRVQVAVKHLH
IHTPLLDSERKDVLREAEILHKARFSYILPILGICNEPEFLGIVTEYMPNGSLNELLHRK
TEYPDVAWPLRFRILHEIALGVNYLHNMTPPLLHHDLKTQNILLDNEFHVKIADFGLSKW
RMMSLSQSRSSKSAPEGGTIIYMPPENYEPGQKSRASIKHDIYSYAVITWEVLSRKQPFE
DVTNPLQIMYSVSQGHRPVINEESLPYDIPHRARMISLIESGWAQNPDERPSFLKCLIEL
EPVLRTFEEITFLEAVIQLKKTKLQSVSSAIHLCDKKK--MELSLNIPV--NHGPQEESC
GSS----QLHENSGSPETSRSLPAPQDNDFLSRKAQ------------------------
------------------------------DCYFMKLHHCPGNHSWDSTISGSQRAAFCD
HKT-T----PCSS--AII-------------NPLSTAGNSERLQPGIAQQWIQSKREDIV
NQMTEACLNQSLDALLSRDLIMKEDYELVSTKPTRTSKVRQLLDTTDIQGEEFAKVIVQK
LKDNKQMGLQPYPEILVVSRSPSLNLLQNKSM
>RIP2-gallus
MSGGAG--G-DRAGAISSALPAIPSSKLPDLRFLSRGAYGTVSAARHADWRVPVALKCL-
-QGPLLDSDRNHLLKEAEILHKARFSYILPILGICNEPDFLGIVTEYMANGSLNQLLHGK
DMYPDIAWCLRYRILYEIALGVNYLHNMNPPLLHHDLKTQNILLDDEFHVKIADFGMSKW
RVISMSQSRSETSLPEGGTIVYMPPEDYNPSQKTRASVKHDIYSFAIIIWEVMSRKHPFE
EVINPLQIMYSVSQGHRLDLSETSLSMDIPHRALVIRLIESGWAQNPDERPSFLKCLIDL
EPVLRTFDEIAILEAVILLKRSKSQYESPCIHKSGKKAIVEKTPLNIPL--N--PQEET-
SASIHCDALPLRSVSPDISRLKSLPQCNILSS-GTA--------GEKAEK----------
----T-------I--GFTAVK--QSIKKCADGDSGG-LNSLSCQSADENLSVTQSAKPFV
HPY-S----YLPSSDKNISVASNSSSCMLQTSPISSDFVLETIQQNVAHQWIQSKREEII
DQMTEACLNQSLDALLSRFLLMKEDYELISTKPTRTSKVRQLLDTSDSQGEEFARIIVQK
LKDNKQLGLQPYPDIV--PNSLRL------HL
>XP_015711414.1_Coturnix_japonica
MSGGEG--G-GRADAISSALPAISSSKLPDLRFLSRGAYGTVSAARHADWRVPVALKCL-
-QGPLLDSDRNHLLKEAEILHKARFSYILPILGICNEPDFLGIVTEYMANGSLNQLLHGK
DMYPDIAWCLRYRILYEIALGVNYLHNMNPPLLHHDLKTQNILLDDEFHVKIADFGMSKW
RVISMSQSRSETSLPEGGTIVYMPPEDYNPSQKTRASVKHDIYSFAIIIWEVMSRKHPFE
EVINPLQIMYSVSQGQRLDLSEKSLSMDIPHRALVIRLIESGWAQNPDERPSFLKCLIDL
EPVLRTFDEIAILEAVILLKRSKSQYESRYIHKSGNKVFVEKSSLNIPV--N--PQEET-
SASIHCDALPLRSISPDISRLKSLPQCNILSS----------------------------
------------------------------DADSGA-PHSLSCQTVDENLSVTQSAKPFV
HPC-G----CLLSSDKNISVSSHSSSCMLQTSPISSDFVLETIQQNVAHQWIQCKREEII
DQMTEACLNQSLDALLSRFLLMKEDYELISTKPTRTSKVRQLLDTSDSQGEEFARIIVQK
LKDNKQLGLQPYPDIV--SNSLRL------HL
>KF074077.1_Cuculus_canorus
MSGGDG--G-SRAGTISYALPSIPSSKLPDLRFISRGAYGTVSAARHADWRVPVALKCL-
-QGPLLDSDRNHLLKEAEILHKARFRYILPILGICNESEFLGIVTEYMTNGSLNQLLHGK
DVYPDVAWCLRFRILYEIALGVNYLHNMNPPLLHHDLKTQNILLDDEFHVKIADFGMSKW
RVISMSQSRSETSLPEGGTIIYMPPEDYNPSQKTRASVKHDIYSFAIIMWEVMSRKQPFE
EVINPLQIMYSVSQGQRLDLSEESLSVDVPHRALIIRLIESGWAQNPDERPSFLKCLIDL
EPVLRTFEEIDMLEAVILLKRSKSLYESRCLYNSEKKADVEQISLNIPL--N--SQEETY
SASIQCDALPLRSISSDISRLESVPKCNILSS-GRD--------THDFIVLF--------
---EH-------R--SNTTLF--FFYFFFKDGNSGG-VDPLSSLSTDENISVSQSAKLLV

HPH-R----YFPSSDKNISGASNAASCVLRSSPTPSDFVLETIQQNVAHQWIQSKREEII
DQMTEACLNQSLDALLSRFLLMKEDYELISTKPTRTSKVRQLLDTSDSQGEEFARIIIQK
LKDNKQLGLQPYPDVV--SNSLRL------HL
>XP_005150905.1_Melopsittacus_undulatus
MSGSDD--G-GQAGAISYALPSISSNKLPDLRFISRGAYGTVSAARHADWRVPVALKCL-
-QGPLLDSDRNHLLKEAEILHKARFSYILPILGICNEPEFLGIVTEYMTNGSLNQLLHGK
DLYPDIPWCLRFRILYEIALGVNYLHNMNPPLLHHDLKTQNILLDDEFHVKIADFGMSKW
RVISMSQSRSETSLPEGGTIIYMPPEDYNPSQKTRASVKHDIYSYAIIMWEVMSRKQPFE
EVINPLQIMYSVSQGQRLDLSEESLSMDIPHRVIIIKLIESGWAQNPDERPSFLKCLIDL
EPVLRTFDEIAMLEAVILLKRSKSLYESQCIYKSGKQADAEQMSLNIPL--N--PHEETY
SGSIQCDALPLRSISPDISRLKSVPMCNILSS----------------------------
------------------------------GGSSGD-LQSLSSQSTDENISVTQSAKLLV
HPY-S----YSPSSDKSISDARNPASCMLRSSPVPSAFVLETIQQNVAHQWIQSKREEII
DQMTEACLNQSLDALLARFLLMKEDYELISTKPTRTSKVRQLLDTSDSQGEEFARIIIQK
LKDNKQLGLQPYPDIV--PNSLRL------HL
>XP_010181516.1_Mesitornis_unicolor
MSGEDG--G-GRAGAICYALPSIPSNKLPDLRFISRGAYGTVSTARHADWRVPVALKCL-
-QGPLLDSDRNHLLKEAEILHKARFSYILPILGICNEPEFLGIVTEYMTNGSLNQLLHGK
DVYPDIPWCLRFRILYEIALGVNYLHNMNPPLLHHDLKTQNILLDDEFHVKIADFGMSKW
RVISMSQSRSETSLPEGGTIIYMPPEDYNPSQKTRASVKHDIYSYAIIMWEVMSRKQPFE
EVINPLQIMYSVSQGQRLDLSEESLSMEIPHRALIIRLIGSGWAQNPDERPSFLKCLIDL
EPVLRTFDEIAMLEAVILLKRSKSQYESRCIYKSRNKADVEQISLNIPL--N--PQEETY
SASIQCDALPLRSISPDISSLKSVPKCNILST----------------------------
------------------------------DANSGD-LHSLSSQSTDENMSVTQSAKLLV
HSC-S----YFPSSDKSISDASNSSPCMLRSSPVPSDFVLETMQQNIAHQWIQSKREEII
DQMTEACLNQSLDALLSRFLLMKEDYELISTKPTRTSKVRQLLDTSDSQGEEFARIIIQK
LKDNKQLGLQPYPNIV--SNSLRL------HL
>XP_014820946.1_Calidris_pugnax
MSGGGA--EEIRAGSISYSLPSIPSNKLPDLRFISRGVYGTVSSARHADWRVPVALKCL-
-QGPLVDSHREYLLKEAEILHKARFSYILPILGICNEPEFLGIVTEYMTNGSLNQLLHGK
DVYPDLPWCLRFRILYEIALGVNYLHNMNPPLLHHDLKTQNILLDDEFHVKIADFGMSKW
RVISMSQSRSETSLPEGGTIIYMPPEDYHPSQKTRASVKHDIYSYAIIMWEVMSRKQPFE
EVINFWQIMYSVSQGKRLDLSEESLSMDIPHRDLIIKLIGSGWAQNPDERPSFLKCLIDL
EPVLRTFDEIAMLEAVLLLKRSKSLYESRCIYKSGKKTDVEQISLNIPL--N--PQEETY
SGSLQCEALPLRSISPDMSRLKSIPKCNILSS-GTSAVKTDETIDFTALKQG--------
----I-------K--KC-AEVLQTWRYSGRGENSEG-LESLSSQSTDENISVTQSAKPLM
QTY-S----NFPSSGKSVSDASNSASCVRQSSPIPSDLVLETIQHNIAHQWIQSKREEII
DQMTEACLNQSLDALLSRFLLMKEDYELISTKPTRTSKVRQLLDTSDSQGEEFAKIIIQK
LKDNKQLGLQPYPDIV--SNSLRL------HL
>XP_014726238.1_Sturnus_vulgaris
MSGGDG--GRGRAGALSYGLPSIPSSKLPDLRFISRGAYGTVSAARHADWRVPVALKCL-
-QGPLLDSDRNHLLKEAEILHKARFTYILPILGICNEPEFLGIVTEYMTNGSLNQLLHGK
DIYPDIPWCLRFRILYEIALGVNYLHNMNPPLLHHDLKTQNILLDDEFHVKIADFGMSKW
RVISMSQSRSESSLPEGGTIIYMPPEDYNPSQKTRASVKHDIYSYAVIIWEVMSRKQPFE
EVINPLQIMYSVSQGQRLDLSEGSLSMDIPHRGLIIRLMESGWAQNPDERPSFLKCIIDL

EPVLRTFDEIAVLEAVILLKRSKSLYESQSIYRSGKKANVEQISLNIPL--N--PEKPTY
SSSIQCDALPLRSISPDISKFKSIPQYNILSS----------------------------
------------------------------DGNSGC-LHSLSSQSTDGNLSVTQSAKLLV
HPY-S----TVLSSDKSISDASGSASHVLRSSPIPSDFVLETTQQNVAHQWIQSKREQII
DQMTEACLNQSLDALLSRFLLMKEDYELISTKPTRTSKVRQLLDTSDSQGEEFARIIVQK
LKDNKQLGLQPYPDIV--SNSQRL------HL
>XP_017681969.1_Lepidothrix_coronata
MSSGDG--GEGRAGAISYALPSIPSNKLPDLRFISRGAYGTVSAARHADWRVPVALKCL-
-QGPLLDSDRNHLLKEAEILHKARFSYILPILGICNEPEFLGIVTEYMTNGSLNQLLHGK
DIYPDIPWCLRFRTLYEIALGVNYLHNMNPPLLHHDLKTQNILLDDEFHVKIADFGMSKW
RVISMSQSRSESSLPEGGTIIYMPPEDYNPSQKTRASVKHDIYSYAIIMWEVMSRKQPFE
EVINPLQIMYSVSQGQRLDLSEESLSMDIPHRVLIIRLMESGWAQNPDERPSFLKCLIDI
EPVLRTFDEIAMLEAVILLKRSKSLYESQGIYKSAKKANVEQISLNIPL--N--PQKETY
SGSIQCDALPLRSISPDISKLKSIPQCNILSS----------------------------
------------------------------DGNSGC-LYSLSSQSMDDNISVTQSAKLLV
HPCTS----NVPSSDRSISDASKSASCILQSPLIPSDFVLETIQQNVAHQWIQSKREEII
DQMTEACLNQSLDALLARFLLMKEDYELISTKPTRTSKVRQLLDTSDSQGEEFARIIVQK
LKDNKQLGLQPYPDIV--SNSQRL------HP
>KQK75542.1_Amazona_aestiva
MSGGDG--G-GRAGAISYALPSIPSNKLPDLRFISRGAYGTVSAARHADWRVPVALKCL-
-QGPLLDSDRNHLLKEAEILHKARFSYILPILGICNEPEFLGIVTEYMTNGSLNQLLHGK
DLYPDIPWCLRFRILYEIALGVNYLHNMNPPLLHHDLKTQNILLDDEFHVKIADFGMSKW
RVISMSQSRSETSLPEGGTIIYMPPEDYNPSQKTRASVKHDIYSYAIIMWEVMSRKQPFE
EVINPLQIMYSVSQGQRLDLSEESLSMDVPHRVIIIKLIESGWAQNPDERPSFLKCLIDL
EPVLRTFDEIAMLEAVILLKRSKSLYESRCIYKSGKQADAEQISLNIPL--N--PHEETY
SGSIQCDALPLRSISPDISRLKSAPKCNVLSS----------------------------
------------------------------DGSSGG-VXSLSSQSTDENISVTQSAKLLV
HPX-S----CSPSSDKNISDATNPASCILRSSPVPSAFLLETIQQNVAHQWIQSKREEII
DQMTEACLNQSLDALLARFLLMKEDYELISTKPTRTSKVRQLLDTSDSQGEEFARIIIQK
LKDNKQLGLQPYPDIV--PNSLRL------HL
>XP_005042333.1_Ficedula_albicollis
MSGGD-----GRAGALSYGLPSIPSSKLPDLRFISRGAYGTVSAARHADWRVPVALKCL-
-QGPLLDSDRNHLLKEAEILHKARFTYILPILGICNEPEFLGIVTEYMTNGSLNQLLHGK
DEYPDIPWCLRFRILYEIALGVNYLHNMNPPLLHHDLKTQNILLDDEFHVKIADFGMSKW
RVISMSQSRSESSLPEGGTIIYMPPEDYNPSQKTRASVKHDIYSYAVIMWEVMSRKQPFE
EVINPLQIMYSVSQGQRLDLSEGSLSMDIPHRGLIIRLMESGWAQNPDERPSFLKCLIDL
EPVLRTFDEIALLEAVILLKRSKSLYESQSIYKSGKKANMEQMSLNIPL--N--SEKPTY
SSSIQCDPLPLRSISPDTSKFKSIPQCNILSS----------------------------
------------------------------DGNSGC-LHSLSSQSMDGNLSVTQSAKLLV
HPY-G----TVLSSDKSNSDASSSASCVLRSSPIPSDFVLETIQQNVAHQWIQSKREQII
DQMTEACLNQSLDALLSRFLLMKEDYELISTKPTRTSKVRQLLDTSDSQGEEFARIIIQK
LKDNKQLGLQPYPDIV--SNSQRL------HL
>XP_005479583.1_Zonotrichia_albicollis
MSGGDGGGG-GRAGALSYGLPSIPSSKLPDLRFISRGAYGTVSAARHADWRVPVALKCL-
-QGPLLDSDRNHLLKEAEILHKARFSYILPILGICNEPEFLGIVTEYMTNGSLNQLLHGK

DVYPDIPWCLRFRILYEIALGVNYLHNMNPPLLHHDLKTQNILLDDEFHVKIADFGMSKW
RVISTSQSRSESSLPEGGTIVYMPPEDYNPSLKTRASVKHDIYSYAVIIWEVMSRKQPFE
EVINPLQIMYSVSQGQRLDLSEGSLSMDIPHRGLIIRLMESGWAQNPDERPSFLKCLIDL
EPVLRTFDEIAMLEAVLLLKRSKSLYESQSIYKSGKKGNVEQIPLNIPL--N--PEKPTY
SSSIECDPLPLRSIPPDTSKFKSIPQCNILSS----------------------------
------------------------------DGNSGC-LHSLSSQSTDENLSVTQSAKLLV
HPY-S----AGLSPARGISDAPSSASHVLRSSPIPPDFVLETMQKNVAHQWIQSKREEII
DQMTEACLNQSLDALLSRFLLMKEDYELISTKPTRTSKVRQLLDTSDSQGEEFARIIVQK
LKDNKQLGLQPYPDIV--SNSQRL------HL
>XP_005517100.1_Pseudopodoces_humilis
MSGGDA--GGGRTGTLSYGLPSIPSSKLPDLRFISRGAYGTVSAARHADWRVPVALKCL-
-QGPLLDSDRNHLLKEAEILHKARFSYILPILGICNEPEFLGIVTEYMANGSLNQLLHGK
DVYPNIPWCLRFRILYEIALGVNYLHNMNPPLLHHDLKTQNILLDDEFHIKIADFGMSKW
RVISMSQSRSESSLPEGGTIIYMPPEDYNPSQKHRASVKHDIYSYAVIMWEVMSRKQPFE
EVINPLQIMYSVSQGQRLDLSEGSLPMDIPHRGLIIRLMESGWAQNPDERPSFLKCLIDL
EPVLRTFDEIAMLEAVILLKRSKSLYESQSIYKSGKKANVEQISLNIPL--N--PEKPMY
YSSIQCDALPLRSISPDTSKFKSIPQCNILSS----------------------------
------------------------------DGDSGC-LHSLSSQSMDGNLSVTQSARPLV
HPN-S----TVLSSDKNISEASSSASSVLTSSPIPSDFVLETIQQNVAHQWIQSKREEII
DQMTEACLNQSLDALLSRFLLMKEDYELISTKPTRTSKVRQLLDTSDSQGEEFARVIVQK
LKDNKQLGLQPYPDIV--SNSQRL------HL
>KFP59105.1_Cathartes_aura

-------SDRNHLLKEAEILHKARFSYILPILGICNEPEFLGIVTEYMTNGSLNQLLHGK
DVYPDIPWCLRFRILYEIALGVNYLHNMNPPLLHHDLKTQNILLDDEFHVKIADFGMSKW
RVISMSQSRSETSLPEGGTIIYMPPEDYNPSQKTRASVKHDIYSYAIIMWEVMSRKQPFE
EVINPLQIMYSVSQGQRLDLSEESLSMDIPHRVLIIRLIRSGWAQNPDERPSFLTCLIDL
EPVLRTFDEIAMLEAVILLKRSKSLYESRCIYKSRKKADVEQISLNIPL--N--PQEETY
SGSIQCDALPLRSISPDISRLKSVPKCNILSS-GSFIMENKTDILLTLSKLD--------
---AI-------V--SC-DDLFPGSCWQEMNGNSGG-LHSLSSQSTDENISVTQSAKLLV
HPY-S----YSPSSDKSISDASNSASCMLRSSPIPSDFVLETIQQNVAHQWIQSKREEII
DQMTEACLNQSLDALLSRFLLMKEDYELISTKPTRTSKVRQLLDTSDSQGEEFARIIIQK
LKDNKQLGLQPYPDIV--PNSLRL------HL
>EOA94323.1_Anas_platyrhynchos

-------SDRNHLLKEAEILHKARFSYILPILGICNEPEFLGIVTEYMTNGSLNELLHGK
DMYPDVPWCLRYRILYEIALGVNYLHNMNPPLLHHDLKTQNILLDDEFHVKIADFGMSKW
RVISMSQSRSETSLPEGGTIIYMPPEDYNPSQKTRASVKHDIYSYAIIMWEVMSRKQPFE
EVINPLQIMYSVSQGQRLDLSEESLSMDIPHRAFVIRLIVSGWAQNPDERPSFLKCLIDL
EPVLRTFDEIAMLEAVILLKRSKSRYESQCIHKSGKKTDVEKTSLNIPL--N--PQEETG
SASIHCDALPLRSISPDISRLKSIPKYNILSS-GAA--------SH--------------
-----------------------FMTCFCTDGDSGS-LHSLSCQSVDENLSVTQSAKLFV
HPY-S----YLPSSDKSISVPSNSSSSVLRTSPIPSDFVLETMQQNVAHQWIQSKREEII
DQMTEACLNQSLDALLSRFLLMKEDYELISTKPTRTSKVRQLLDTSDSHGEEFSRIIIQK
LKDNKQLGLQPYPDIV--SNSLRL------HL

>KFP60755.1_Cariama_cristata

-------SDRKHLLKEAEILHKARFSYILPILGICNEPEFLGIVTEYMANGSLNQLLHGK
DLYPDIPWCLRFRILYEIALGVNYLHNMNPPLLHHDLKTQNILLDDEFHVKIADFGMSKW
RVISMSQSRSETSLPEGGTIIYMPPEDYNPSQKTRASVKHDIYSYAIIMWEVMSRKQPFE
EVINPLQIMYSVSQGQRLDLSEESLSMDIPHRVVIIRLIGSGWAQNPDERPSFLKCLIDL
EPVLRTFDEIAMLEAVILLKRSKSLYESRCIYKSGKQADVEQISLNIPL--N--PQEETY
SGSIQCDALPLRSISPDGARLKSVPTCNILST-GRY--------THGFVKLD--------
---FC-------L--CQKRDV--FCKFSTLDGNSGG-LHSLSSQSTDENISVTQSAKLLV
HPY-S----YFPSSDKSISDASNSASCMLRSSPVPSDFVLETIQQNVAHQWIQSKREEII
DQMTEACLNQSLDALLSRFLLMKEDYELISTKPTRTSKVRQLLDTSDSQGEEFARIIVQK
LKDNKQLGLQPYPDIV--SNSLKL------HL
>KFQ90254.1_Phoenicopterus_ruber

-------SDRNHLLKEAEILHKARFSYILPILGICNEPEFLGIVTEYMTNGSLNQLLHGK
DVYPDIPWCLRFRILYEIALGVNYLHNMNPPLLHHDLKTQNILLDDEFHVKIADFGMSKW
RVISMSQSRSETSLPEGGTIIYMPPEDYNPSQKTRASVKHDIYSYAIIMWEVMSRKQPFE
EVINPLQIMYSVSQGQRLDLSEESLSMDIPHRVLIIRLIGSGWAQNPDERPSFLKCLIDL
EPVLRTFDEIVMLEAVILLKRSKSLYESRCIYKSGKKADVEQISLNIPL--N--PQEEAY
SASIQCDALPLRSISPDISRLKSVPKCNILSS-GRD--------THDFVKLD--------
---HR-------L--CQKGDV--FCKLSALDGNSGG-QHSLSSQSTDENISVTQSAKLLV
HPY-S----YFPSSDKSISEASNSASYMLRSSPVPSDFVLETIQQNVAHQWIQSKREEII
DQMTEACLNQSLDALLSRFLLMKEDYELISTKPTRTSKVRQLLDTSDSQGEEFARIIIQK
LKDNKQLGLQPYPDIV--SNSLRL------HL
>KFQ63971.1_Pelecanus_crispus

-------SDRSHLLKEAEILHKARFSYILPILGICNEPEFLGIVTEYMTNGSLNQLLHGK
DVYPDIPWCLRFRILYEIALGVNYLHNMNPPLLHHDLKTQNILLDDEFHVKIADFGMSKW
RVISMSQSRNETSLPEGGTIIYMPPEDYNPSQKTRASVKHDIYSYAIIMWEVMARKQPFE
EVINPLQIMYSVSQGQRLDLSEESLSMDIPHRVLVIRLIGSGWAQNPDERPSFLKCLIDL
EPVLRTFDEIAMLEAVILLKRSKSLYESRCIYKSGKKADVEQIALNIPL--N--PQEETY
SGSIQCDALPLRSISPDISRLKSVPKCNILSS-ESL--------NLMFLQLN--------
---FH------------------FYYSSLLDGNSGG-LHSLSSQSTDENISVTQGAKLLV
HPY-S----YCPSSDKSISDASNSASCMLRSSPTPSDFVLETIQQNVAHQWIQSKREEII
DQMTEACLNQSLDALLSRFLLMKEDYELISTKPTRTSKVRQLLDTSDSQGEEFARIIIQK
LKDNKQLGLQPYPDIV--SNSLRL------HL
>KFM04929.1_Aptenodytes_forsteri

-------SDRNHLLKEAEILHKARFSYILPILGICNEPEFLGLVTEYMTNGSLNQLLHGK
DVYPDIPWCLRFRILYEIALGVNYLHNLNPPLLHHDLKTQNILLDNEFHVKIADFGMSKW
RVISMSQSRSETSLPEGGTIIYMPPEDYNPSQKTRASVKHDIYSYAIIMWEVMSRKQPFE
EVINPLQIMYSVSQGQRLDLSEESLSMDIPHRVLIIRLIGSGWAQNPDERPSFLKCLIDL
EPVLRTFDEIAMLEAVILLKRSKSLYESRCIYKSGKKADVEQISLNIPL--N--PQEETY
SGSIQCDALPLRSISPDISRLKSVPKCNILSS-GSFIMESKTDILLSLPKLD--------
---AI-------V--SG-ENLFPGSCWQEMNGNSGG-LHSLSSQSTDENISVTQSAKLLV

```
DPY-S----YFPSSDKSISDASNSASCMLRSSPIPSDFVLETVQQNVAHQWIQSKREEII
DQMTEACLNQSLDALLSRFLLMKEDYELISTKPTRTSKVRQLLDTSDSQGEEFARIIIQK
LKDNKQLGLQPYPDIV--SHSLRL------HL
>KFQ74688.1_Phaethon_lepturus
------------------------------------------------------------
-------SDRNHLLKEAEILHKARFSYILPILGICNEPEFLGIVTEYMTNGSLNQLLHGK
DVYPDIPWCLRFRILYEIALGVNYLHNMNPPLLHHDLKTQNILLDDEFHVKIADFGMSKW
RVISMSQSRSETSLPEGGTIIYMPPEDYNPSQKTRASVKHDIYSYAIIMWEVMSRKQPFE
EVINPLQIMYSVSQGQRLDLSEESLSVDIPHRVLIIRLIGSGWAQNPDERPSFSKCLIDL
EPVLRTFDEIAMLEAVILLKRSKSLYESQCICKSGKKADVEQISLNIPL--N--PQEEIY
SASIQCDALPLRSISPDISRLKSVPKCNILSS-VSD--------GCILFEHG--------
---SN-------I--TC-FFF--FFAFFFLDGNSGG-LHSLSSQSMDENISVTQSAKLLV
HPY-S----HFPSSDKSISDASNSASCMLRSSPIPSDFVLETIQQNVAHQWIQSKREEII
DQMTEACLNQSLDALLSRFLLMKEDYELISTKPTRTSKVRQLLDTSDSQGEEFARIIIQK
LKDNKQLGLQPYPDIV--SNSLRL------HL
>KFQ07057.1_Haliaeetus_albicilla
------------------------------------------------------------
-------SDRKHLLKEAEILHKARFSYVLPILGICNEPEFLGIVTEYMTNGSLNQLLHGK
DVYPDIPWCLRFRILYEIALGVNYLHNMNPPLLHHDLKTQNILLDDEFHVKIADFGMSKW
RVISMSQSRSETSLPEGGTIIYMPPEDYNPSQKTRASVKHDIYSYAIIMWEVMSRKQPFE
EVINPLQIMYSVSQGQRLDLSEESLSMDIPHRVLIIRLIGSGWAQNPDERPSFLKCLIDL
EPVLRTFDEIAMLEAVILLKRSKSLYESRCICKSGKKADVEQISLNIPL--N--PQEETY
SGSIQCDALPLRSMSPDVSRLKSVPKCNILSS-ELY--------LT-YIRIL--------
---FF-------L--SH-KIL--HCRIIYLDGNSGV-LHSLSSQSTDENISVTQSAKLLE
HPY-S----YFPSSDKSISGASNSASCMLRSSPIPSDFVLETIQQNVAHQWIQSKREEII
DQMTEACLNQSLDALLSRFLLMKEDYELISTKPTRTSKVRQLLDTSDSQGEEFARIIIQK
LKDNKQLGLQPYPDIV--SNSLRL------HL
>KFW05819.1_Eurypyga_helias
------------------------------------------------------------
-------SDRNHLLKEAEILHKARFTYILPILGICNEPEFLGIVTEYMTNGSLNQLLHGR
DVCPDIPWCLRFRILYEIALGVNYLHNMNPPLLHHDLKTQNILLDDEFHVKIADFGMSKW
RVISMSQSRSETSLPEGGTIIYMPPEDYNPSQKTRASVKHDIYSYAIIMWEVMSRKQPFE
EVINPLQIMYSVSQGQRLDLSEESLSMDIPHRELIIRLIESGWAQDPDERPSFLKCLIDL
EPVLRTFEEIAMLEAVILLKRSKSLYESRCIYKSGKRADVKQISLNIPL--N--PQEETC
SASIQCDALPLRSISPDISRLKSVSRCNILSS-ACF--------FFFFPP----------
-------------------PS--LPFLFFKDGNSGG-LQSLSSQSMDENISVTQSAKLLV
HPY-S----YFPSSDKSISDASNSASCMLRSSPVPSDFVLETIHQNVAHQWIQSKREEII
DQMTEACLNQSLDALLSRFLLMKEDYELISTKPTRTSKVRQLLDTSDSQGEEFARIIIQK
LKDNKQLGLQPYPDVV--SNSLRL------HL
>KFW67401.1_Pygoscelis_adeliae
------------------------------------------------------------
-------SDRNHLLKEAEILHQARFTYILPILGICNEPEFLGLVTEYMTNGSLNQLLHGK
DVYPDIPWCLRFRILYEIALGVNYLHNMNPPLLHHDLKTQNILLDNEFHVKIADFGMSKW
RVISMSQSRSETSLPEGGTIIYMPPEDYNPSQKTRASVKHDIYSYAIIMWEVMSRKQPFE
EVINPLQIMYSVSQGQRLDLSEESLSMDIPHRVLIIRLIGSGWAQNPDERPSFLKCLIDL
```

```
EPVLRTFDEIAMLEAVILLKRSKSLYESRCIYKSGKKADVEQISLNIPL--N--PQEETY
SGSIQCDALPLRSISPDISRLKSVPKCNILSS-ESL--------DLIFLLLN--------
---F---------------HFYYSSLLGQLDGNSGS-LHSLSSQSTDENISVTQSAKLLV
DPY-S----YLPSSDKSISNASNSASCMLRSSPIPSDFVLETVQQNVAHQWIQSKREEII
DQMTEACLNQSLDALLSRFLLMKEDYELISTKPTRTSKVRQLLDTSDSQGEEFARIIIQK
LKDNKQLGLQPYPDIV--SHSLRL------HL
>KFU96217.1_Chaetura_pelagica
------------------------------------------------------------
-------SDRNHLLKEAEILHKARFSYILPILGICNEPEFLGIVTEYMTNGSLNQLLHGK
DVYPEIPWCLRFRILYEIALGVNYLHNMNPPLLHHDLKTQNILLDDEFHVKIADFGMSKW
RVISMSQSRSETSLPEGGTIIYMPPEDYNPSQKTRASVKHDIYSYAIIMWEVMSRKRPFE
EVINPLQIMYSVSQGQRLDLSEESLSMDIPHRVLIVRLIASGWAQNPDERPSFLKCLLEL
EPVLRTFDEIAMLEAVILLKRSKSQYESQCIYKSGKKTDVKQISLNIPL--N--PQEETC
SASIQCDALPLRSISPDVSTFKSVPKCNILSS-GGS--------LFM-P-----------
-----------------KRDV--FCKISALDGDSEV-LHSLSSQSTDENTSITQSAKLIV
HPY-S----SFPSSDKCISDASNSASYMLRSSPVPSDFASETIQQNVAHQWIQSKREEII
DQMTEACLNQSLDALLSRFLLMKEDYELISTKPTRTSKVRQLLDTSDSQGEEFARIIIQK
LKENKQLGLQPYPDIV--SNSQRL------HL
>KFR17723.1_Opisthocomus_hoazin
------------------------------------------------------------
-------SDRNHLLKEAEILHKARFSYILPILGICNEPGFLGIVTEYMTNGSLNQLLHGK
DVYPDIPWCLRFRILYEIALGVNYLHNMNPPLLHHDLKTQNILLDDEFHVKIADFGMSKW
RVISMSQSRSETSLPEGGTIIYMPPEDYNPSQKTRASVKHDIYSYAVIMWEVMSRKQPFE
EVINPLQIMYSVSQGQRLDLSGESLSMDIPHRALIIRLIGNGWAQNPDERPSFLKCLIDL
EPVLRTFDEIAMLEAVILLKRSKSLYESQCIYKSGKKAGVEQVSLNIPL--N--SQEETY
SGSLQCDALPLRSISSGTSRLQSVPKCNILSS-D---------ILLTLPKLD--------
---AI-------I--SV-ENLFPGRCWQEMNGNSGS-LHSLSSQSTDENISVTQSAKLLM
HPY-S----YFPSSDKSISDTSNSSPCMLRSSPIPADFALETIQHNVAHQWIQSKREEII
DQMTEACLNQSLDALLARFLLMKEDYELISTKPTRTSKVRQLLDTSDSQGEEFARIIIQK
LKDNKQLGLQPYPDIV--SNSLRL------HL
>KFR01688.1_Nipponia_nippon
------------------------------------------------------------
-------SDRNHLLKEAEILHKARFSYILPILGICNEPEFLGIVTEYMTNGSLNQLLHGK
DVYPDIPWCLRFRMLYEIALGVNYLHNMNPPLLHHDLKTQNILLDDEFHVKIADFGMSKW
RVISMSQSRSETSLPEGGTIIYMPPENYNPSQKTRASVKHDIYSYAIIMWEVMSRKQPFE
EVINPLQIMYSVSQGQRLDLSEESLSMDIPHRVLIIRLIGSGWAQNPDERPSFLKCLIDL
EPVLRTFDEIAMLEAVILLKRSKSLYESRCIYKSGKKADVEQISLNIPL--N--PQEETY
SGSIQCDALPLRSISPDISRLKSIPTCNILSS-ESL--------DFIFLLLN--------
---F---------------HFYYSSLLGQLDGNSGS--HSLSSQSMDENISVTQGAKLLV
HPY-S----YCPSPNKSISDASNSASCMLRSSPIPSDFVLETVQQNVAHQWIQSKREEII
DQMTEACLNQSLDALLSRFLLMKEDYELISTKPTRTSKVRQLLDTSDSQGEEFARIIIQK
LKDNKQLGLQPYPDAV--SNSLRL------HL
>KFP01549.1_Calypte_anna
------------------------------------------------------------
-------SDRNHLLKEAEILHKARFSYILPILGICNEPEFLGIVTEYMTNGSLNELLHGK
```

DVYPDIPWCLRFRILYEIALGVNYLHNMDPPLLHHDLKTQNILLDDEFHVKIADFGMSKW
RVISMSQSRSETSLPEGGTIIYMPPEDYNPSQKTRASVKHDIYSYAIIMWEVMSRKQPFE
EVTNPLQIMYSVSQGQRLDLSEESLSMDIPHRALVLRLIGSGWAQNPDERPSFLKCLLEL
EPVLRTFDEIAMLEAVVLLKRSKSLYESRCIYKIGKKADVQQISLNIPL--N--PQEETC
SASIQCDAFPLRSISPVISRFKSVSKSNILSS-SRD--------NV-YTRFL--------
---FF-------L--SQ-EIL--HYRISCLDGNSEG-LHSLSNQSTDENTSVTQSARLLG
HPY-S----SLPSSDKCISNASNSASCGLPSSPLPSDFLLETIQQNIAHQWIQSKREEII
DQMTEACLNQSLDALLSRFLLMKEDYELISTKPTRTSKVRQLLDTSDSQGEEFARIIIQK
LKDNKQLGLQPYPDVI--PNSLKL------HL
>KFP88042.1_Acanthisitta_chloris
---CL-
-FACFLLSDRDHLLKEAEILHKARFSYILPILGICNEPEFLGIVTEYMTNGSLNQLLHGK
DVYPDIPWCLRFRILYEIALGVNYLHNMNPPLLHHDLKTQNILLDDEFHVKIADFGMSKW
RVISMSQSRSESSLPEGGTIIYMPPEDYNPSQKTRASVKHDIYSYAVIMWEVMSRKQPFE
EVINPLQIMYSVSQGQRLDLSEESLPIDIPHRMLVIRLMESGWAQNPDERPSFLKCLIDL
EPVLRTFDEIAMLEAVILLKRSKSLYESRCIYKSGKKANVDQISLNIPL--N--PPKETY
SGSIQCDALPLRSISPDISKIKSVPQCNILSP-GEI--------LLILYKFKLLLWLYTP
VVTFHFLSRKLGL--SQKIDV--LCKLSALDGSSVG-LHSLSSQSTDENISVTQSAKLLV
HPY-S----NAPSSDKSISDASNSASCMLQSSPIPADFILETIQQNVAHQWIQSKREEII
DQMTEACLNQSLDALLSRYLLMKEDYELISTKPTRTSKVRQLLDTSDSQGEEFARIIVQK
LKDNKQLGLQPYPDIV--SNSQRL------HL
>KFO87475.1_Buceros_rhinoceros_silvestris

-------SDRNHLLKEAEILHKARFSYVLPILGICNEPEFLGIVTEYMPNGSLNQLLHGK
DVYPDISWCLRFRILYEIALGVNYLHNMNPPLLHHDLKTQNILLGDEFHVKIADFGMSKW
RVISVSQSRSETSLPEGGTIIYMPPEDYNPSQKTRASVKHDIYSYAVIMWEVMSRKQPFE
EVINPLQIMYSVSQGQRLDLSEESLSMDIPHRLLIIRLIGSGWAQNPDERPSFLKCLIDL
EPVLRTFDEITMLEAVIRLKRSKSLYESQHINKSGKKADVEQITLNIPL--N--LQEETY
LGSIQCDALPLRSISPEISRPKSVSKCNILSS-DVL--------LT-LPKLH--------
---AI-------V--SA--RS--FFSLFFKDGSSEG-PHSLSSKSTDENISVTQSAKLLV
HPY-G----YFPSSDKSISDGSNSASCMLRSSPTPSDFVLETLQQNVAHQWIQSRREEII
DQMTEACLNQSLDALLARFLLMKEDYELISTKPTRTSKVRQLLDTSDSQGEEFARIIIQK
LKDNKQLGLQPYPDII--SNSLRL------HL
>KFP33539.1_Colius_striatus

-------SDREHLLKEAEILHKARFSYILPILGICNEPEFLGIVTEYMTNGSLNQLLHGK
DIYPDVPWCLRFRILYEIALGVNYLHNMNPPLLHHDLKTQNILLDDEFHVKIADFGMSKW
RIISMSRSRSETSLPEGGTIIYMPPEDYNPSQKTRASVKHDIYSYAILMWEVMSRKQPFE
EVINPLQIMYSVSQGQRLDLSEKSLSMDIPHRVLIIRLIGSGWAQNPDERPSFLKCLIDL
EPALRTFDEIAMLEAVTLLKRSKTLYESPCIYKSGKNTDVEQISLNIPL--S--PQEETC
SGSVQCDALPLRSISPDMSRVKSICKCNILSS-ANW--------VFFC------------
------------------------FFFIFEDVNSGC-LHSLSSQSMDENISVTQRAKLLG
HPY-G----CFHSSDKSSSDASSSAPCRLQSSPIPSDFVLETIQQNVAHQWIQSKREEII
DQMTEACLNQSLDALLSRFLLMKEDYELISTKPTRTSKVRQLLDTSDSQGEEFARIIIQK
LKDNKQLGLQPYPDIV--SNSLTL------HL

>KF057232.1_Corvus_brachyrhynchos

-------SDRNHLLKEAEVLHKARFSYILPILGICNEPEFLGIVTEYMTNGSLNQLLHGK
DIYPDIPWCLRFRILYEIALGVNYLHNMNPPLLHHDLKTQNILLDDEFHVKIADFGMSKW
RVISMSQSRSESSLPEGGTIIYMPPEDYNPTQKTRASVKHDIYSYAVIMWEVMSRKQPFE
EVINPLQIMYSVSQGQRLDLSEGSLSMDIPHRGLIIRLMESGWAQNPDERPSFLKCLIDL
EPVLRTFDEIAMLEAVILLRRSKSVYESQCIYKSGKKANMEQISLNIPL--N--PDKPAY
SSSIQCDALPLRSISPDISKLKSIPQCNILSS-GEI--------LLIFRKLG--------
---HS-------M--SE-RNV--FCKLCALDGNSGC-LHSLSSQSTDGNLSVTQSAKVLV
HPY-S----TVLSSDKSISDASSSSSCVLRSSPIPSDFVLETVQQNVAHQWIQSKREEII
DQMTEACLNQSLDALLSRYLLMKEDYELISTKPTRTSKVRQLLDTSDSQGEEFARIIVQK
LKDNKQLGLQPYPDIV--SNSQRL------HL
>XP_010403308.1_Corvus_cornix
MSGGDG--GGGRAGALSYALPSIPSSKLPDLRFISRGAYGTVSAARHADWRVPVALKCL-
-QGPLLDSDRNHLLKEAEVLHKARFSYILPILGICNEPEFLGIVTEYMTNGSLNQLLHGK
DIYPDIPWCLRFRILYEIALGVNYLHNMNPPLLHHDLKTQNILLDDEFHVKIADFGMSKW
RVISMSQSRSESSLPEGGTIIYMPPEDYNPTQKTRASVKHDIYSYAVIMWEVMSRKQPFE
EVINPLQIMYSVSQGQRLDLSEGSLSMDIPHRGLIIRLMESGWAQNPDERPSFLKCLIDL
EPVLRTFDEIAMLEAVILLRRSKSVYESQCIYKSGKKANMEQISLNIPL--N--PDKPAY
SSSIQCDALPLRSISPDISKLKSIPQCNILSS----------------------------
------------------------------DGNSGC-LHSLSSQSTDGNLSVTQSAKVLV
HPY-S----TVLSSDKSISDASSSSSCVLRSSPIPSDFVLETVQQNVAHQWIQSKREEII
DQMTEACLNQSLDALLSRYLLMKEDYELISTKPTRTSKVRQLLDTSDSQGEEFARIIVQK
LKDNKQLGLQPYPDIV--SNSQRL------HL
>KF013799.1_Balearica_regulorum

-------SDRNHLLKEAEILHKARFSYILPILGICNEPEFLGIVTEYMTNGSLNQLLHGK
DVYPDIPWCLRFRILYEIALGVNYLHNMNPPLLHHDLKTQNILLDDEFHVKIADFGMSKW
RVISMSQSRSETSLPEGGTIIYMPPEDYNPSQKTRASVKHDIYSYAIIMWEVMSRKQPFE
EVINPLQIMYSVSQGQRLDLSEESLSMDIPHRVLIVRLIGSGWAQNPDERPSFLKCLIDL
EPVLRTFDEIAMMEAVILLKRSKSLYESRCIYKSGKKADVEQISLNIPL--N--PQEEAY
SGSIQCDALPLRSISPDTSRLKSVPKCNILSS-DEI--------DFTAMKEG--------
----I-------K--KC-AEVLQNCRYSGRDGNSGG-LHSLSTQSMDENISVTQSAKLLV
HPY-S----YFPSSDKSISDASNSASCMLRSSPIPSDFVLETIQQNIAHQWIQSKREEII
DQMTEACLNQSLDALLSRFLLMKEDYELISTKPTRTSKVRQLLDTSDSQGEEFARIIIQK
LKDNKQLGLQPYPDIV--SNSLRL------HL
>KFV81187.1_Struthio_camelus_australis

-------SDRNHLLKEAEILHKARFSYILPILGICNEPEFLGIVTEYMTNGSLNQLLHGK
DVYPDIPWCLRFRILYEIALGVNYLHNMNPPLLHHDLKTQNILLDDEFHVKIADFGLSKW
RVVSMSQSRSETSLPEGGTIIYMPPEDYNPSQKSRASVKHDIYSYAIIMWEVMTRKQPFE
EVINPLQIMYSVSQGQRLDLSKESLSMDIPHRALIIRLIASGWAQNPDERPSFLKCLIDL
EPVLRTFDEIAMLEAVILLKRSKLLYESQCIHKSGKKTHADQTSLNIPLNLN--PQEEMH
SVSIQYDALPLRSIPPDVSTFKSAPKYNILSS-GI---------LLTLPKLD--------
---AV-------V--PA-ENLFLGGCWQEMNGNSGD-LHSSSSQSMDENFSVTQSAKLLV

HPY-S----SSASSDKSISSASN-----LRSSPIPSDFVLETIQHNVAHQWIQSKREEII
DQMTEACLNQSLDALLSRFLLMKEDYELISTKPTRTSKVRQLLDTSDSQGEEFARIIIQK
LKDNKQLGLQPYPDIV--SNSLRL------HL
>KGL77642.1_Tinamus_guttatus

-------SDRNHLLKEAEILHKARFSYILPILGICNEPEFLGIVTEYMTNGSLNQLLHGK
DVYPDIPWCLRFRILYEIALGVNYLHNMNPPLLHHDLKTQNILLDDEFHVKIADFGLSKW
RVVSMSQSRSEMSLPEGGTIIYMPPENYTPSQKTRASVKHDIYSYAIIMWEVMARKQPFE
EVINPLQIMYSVSQGQRLDLSEESLSVDIPHRALVTRLIASGWAQNPDERPSFLKCLIDL
EPVLRTFDEIAMLEAVILLKRSKSLYESRCIHKSGKKTNADQTFLNIPL--NPNPQEEMH
SASIQYDGLPLQSIPPDVSTYKSVPKYNILSS-GRD--------IHDFIS----------
------------------------INSFFLDGNSGD-LHSLSNQSMDENISVTQSAKLLV
HPY-TDSFTCSPSSDRSISSASNSAPCMLRSSPTPPDFVLEAMQPNVAHQWIQSKREEII
DQMTEACLNQSLDALLSRFLLMKEDYELISTKPTRTSKVRQLLDTSDSQGEEFARIIIQK
LKDNKQLGLQPYPDIV--PTSLRL------HL
>KFZ59450.1_Podiceps_cristatus

---FQIADFGMSKW
RVISMSQSRSETSLPEGGTIIYMPPEDYNPSQKTRASVKHDIYSYAIIMWEVMSRKQPFE
EVINPLQIMYSVSQGQRLDLSEESLSMDIPHRVLIIRLIGSGWAQNPDERPSFLKCLIDL
EPVLRTFDEIVMLEAVILLKRSKSLYESRCLYKSGKKADAEQITLNIPL--N--PQEETY
SASIQCDALPLRSISPDISSIKSVPKCNILSS-DLM--------FC-AAKFS--------
---LL-------LFQSS-RST--RFFLSNLNGSSGG-QHSLSSQSTDENISVTQSAKLLV
HPY-S----YFSSSDKNISNASNSASYMLRSSPIPSDFVLETIQQNVAHQWIQSKREEII
DQMTEACLNQSLDALLSRFLLMKEDYELISTKPTRTSKVRQLLDTSDSQGEEFARIIIQK
LKDNKQLGLQPYPDIV--SNSLRL------HL
>KFQ47898.1_Nestor_notabilis

---FQIADFGMSKW
RVISMSQSRSETSLPEGGTIIYMPPEDYNPSQKTRASVKHDIYSYAIIMWEVMSRKQPFE
EVINPLQIMYSVSQGQRLDLSAESLSMGIPHRVIIVRLIESGWAQNPDERPSFLKCLIDL
EPVLRTFDEIAMLEAVILLKRSKSLYESQSIYKSGKQADTEQISLNIPV--N--THEETY
SGSIQCDALPLRSISPDISRLKSVPKCNIFSS-GRD--------THNFVNTA--------
---G---------------SF-VVSYFLFLDGNSGG-LQSLSCQSTDENISVTQSARLLV
HPY-S----CFPSSDKSNSDASNSAPCMLRSSPIPSDFVLETVQQNIAHQWIQSKREEII
DQMTEACLNQSLDALLSRFLLMKEDYELISTKPTRTSKVRQLLDTSDSQGEEFARIIIQK
LKDNKQLGLQPYPDIV--PNSLRL------HL
>KFV95703.1_Fulmarus_glacialis

---FQIADFGMSKW
RVISMSQSRSETSLPEGGTIIYMPPEDYNPSQKTRASVKHDIYSYAIIMWEVMSRKQPFE
EVINPLQIMYSVSQGQRLDLSEESLSMDIPHRVLIIGLIGSGWAQNPDERPSFLKCLIDL

EPVLRTFDEIAMLEAVILLKRSKSLYESRCIYKSEKKANVEQISLNIPL--N--PQEETC
SASIQCDALPLRSISPDISRLKSVPKCNILSS-ESL--------DLIFLLLN--------
---F---------------HFCYSSLLGQLDGNSGD-LHSHSSQSTDENISVTQSAKLLV
HPY-S----YFPSSDQSISDASNSASCMLRSSPIPSDFVLETIQQNVAHQWIQSK**REEII**
DQMTEACLNQSLDALLSRFLLMKEDYELISTKPT**RTSKVR**QLLDTSDSQGEEFARIIIQK
LKDNKQLGLRPYPDIV--SNSLKL------HV
