# Supplementary material for: Structural and Evolutionary Adaptation of NOD-Like Receptors in Birds
Source: Biomed Res Int. 2021 Apr 29;2021:5546170. doi: 10.1155/2021/5546170 (PMC8105094; doi:10.1155/2021/5546170)
Supplement: Supplementary Materials — S1: RIP2 alignment. S2: primers and chicken sequences and ancestral sequences. S3: avian sequences accession number. Supplementary Table 1: sites found to be under positive selection. [file 5546170.f1.zip › S2 Primers and chicken sequences and ancestral sequences.pdf]

Primers

Target gene（bp）	Forward primer (5’-3’)	Reverse primer (5’-3’)
NOD1	CCTCCTCCCGTCCGTCC	TCAGAAGCAAATGATCCG
NLRC3(1~936)	ATGGAAGAGGCTTGGATCAG	CTCCGTCATGCGGTCTAC
NLRC3(676~1507)	CCCCACATCACGGAACCCAAC	GATCGAATATCGCCCGCTTG
NLRC3(1433~2482)	ATTTCTCCCACTTAACCATAC	CCGAGCCGTTGTCTCCTAC
NLRC3(2216~3276)	GCCCATCGGGAGCGAAAGC	TCACACCACCACCACGCAG
NLRP3(1~1024)	ATGGCAGGAGAAGAAAGCACC	AGGCCACATCTGCTTTACTG
NLRP3(676~1782)	TGCCCTCAACAGCGAATGC	GAAACTGCCCAACACGCTC
NLRP3(1423~2202)	CTCTTCGCAGCGATGTTTTAC	TCACCGCAGGACACGCAGC

>NOD1-chicken

MEGHLCANLKTSVEKPLTTSPPSCIALLKVYRELLVSKIRHTQCLIDNLINNEYFSTEDAEIVVQFPTQ
ADKVRKILDLVQSKGEEVSEYFICVLQKVTDAYYELQPWLDEIGYQPSENICSKPVVNTDPVSRYCQKL
RYELGRDSKFVLSYAQREEMLLEEIYSNSIMEMVNFTNESLGEVCQLQALFDDAVGVINEDGETIYVFG
DAGIGKSILLQKIQSLWAKKQLDIGAKFFFHFRCRMFSCFKEDEAVCLKDLLFRYNCYPDQDPTEVFHH
ILQFPHTVLFTFDGFDEIYSNFDLSSVPEMCSPDEPMHPLVLLVSLLRGKLLKGSKKILTARTGTEIQR
NIIRKKVLLRGFSNSNLKEYTAMFFKDVGQRTLVLNQLEANPNLCSLCSVPLFCWIIFKCYEHFHSMFD
SHELPDCSVTLTDVFLLMIEVHLNRSLKTSLLKNNTRSQAEVFRSRKETLLALGKIAYRGMGNSIFIFE
QEEVSSANISEEDLQLGFLRTVKGYSRCDNQSTYEFLHLTLQSFFTALFLVIEERVGNKELLQFFNECS
SAEAAQRTCFRVPWLKKPLTGEDPFRNNEHFHFTNLFLCGLLSRSKEKLFKHLVSPAVIRKKRKTLITY
LGESMKSHLKGVTRSRLPMYNQVQVQPNFVWMLRCLYETQSEKVGKLAAKRMHANYIKLTYCNAYSADC
SAISFVVHHFQKHLALDLDNNNINDYGVKQLLPCFSKLAVIRLSVNQITDHGARILYEELSKYQIVSYL
GLYNNQITDIGAKYVAKLIEECSSLEYVKIGANKITSEGGKCLAQAIQKSTTMFEIGMWGNQVGDEGAK
AFADALRNHPRLTNVSLAFNGITTEGGKSIAEALQHNDSVKIFWLTKNELDDEAAMSFAEMLKINKKLV
HLWLIQNQITAKGVKYLSEALKENTAIKEVCLNGNLISQEEAKAFESEERIICF

>NLRC5-chicken-AEY11256.1

MQPTLQNDDFNLEGAVASIRPQLVEFLSHRLDWLLSASQHFLPTAVLSGLAGVTDHREKVSVLLDLLEK
AGHATWKQFAQCLCMECDLPLEMEILLMSSAGEGNLTQKQEAQTDASDQSSVPRGLTRRRRSSSPISDK
GAKQKRLDSAEKYQRLLVDSICKRYGSRRAAGAAAQEPTQPLAFSQAFVNLVLRQSKASRLKERTDKPR
EDVPSAPEPEECVDTAVRVSDLFGSVVRSGTTKVIFLFGKPGTGKTMLMHRICQKWAEGVLHQFLFTFL
FEFRQLNLLKRKLTLKELLFDLFLQPEDSPDAVFQHLLENAQRTLIIFDGLDEFVGNMDVSSASKGSLN
RSSSMSISELFAELCHGNLLPGCTVLVTSRPKRVPDFLINTVDLLAEVWGFDHEKVEEYVSHYFRHHSF
KEQAIAHLKNNTKLLSMCQIPALCYVVCICLEYLLLKHQTSVELPQTMTQFYIKMLLIFINKQQGEHAG
DEEAQLNSNKKAILGLCDLALKGLEAKKLVFYVGDIPEHVKEFASLHGLLTVFEVKTNSAHPETGYAFV
HLSLQEFFAALCLMISKSVDKSHLKRKLSLKSKWTLRNEAKTGFMESFHIFLSGLSSKECRTFIMLLAE
QSEAWVQDKQDAILQSLKKLAATQLTGPKVIELCHCTFETQGLEVAQHIGSLLNFKYEFKNFRLTPLDM
SALVFVINSGQDVTHLDFAGCPLDTDCLEMLASCRNVEHLSFRSRRFGDDFAAALSKGLGEMGSLKKLE
VTGGSITAAGLTDMVQALSHCLQLEEINLQDNRIQNPDVKTVMELFSRMEKLKKIDLSKNSLSLNAVLL
LAKEFIACQNAAELHVRKDTVIISFSGPSGKVPRSLDLKREQNKECVTPTRHLRLCLQARCLSSQHAKE
IVSILQSCPHLSEVDLSDNKLGDEGCSFLLENLSWISISKQLNLSHNLLSVTGIYSLLKAVNTCQRVVE

VEVSLYHNTAVLRFTEDGEFASPPASREEPLYPTDDQWDDKENQTPVPSKKIRLTYCRFQASDLEKLCA
VLKECGSISELDLSNNYLGDEGLAQLLQFLPNLKTLRSLKLNNNHLSLSSVFCLAQSLCTLEHLETMDL
SLGRMQVVHLTFGDRIRLRRTSRWRRSFLVHPKHVTNGQCFRLRNCTVGPEDVTRLCQMLTQCTQLTEI
DLCGNALNDQSIERLLSFLPHFCQLTLLSIRNNTFSPCCAVLFANSINLCERIRRVEVRSSQNAFLHLR
TSTQSQKTSCRLTDCAIGQRQIEKLCRVLEQHGCLAELDLSRNQLGDEVLRFLLDHLHRVPVTCSLNLS
HNRISQRGVLHLINTFTTSGNTTEVQVSLCSKATLIIKMTSRDDPRKILRLTECSFQPEHLEKLCLVLE
NCTNLTECISSNNNVTVDAAGGLLRSLSKTPGPLKISIEEPWVCKLSVTSLLELAVQAHGNITAIMICK
EKNLFQLGVRLPHCLEKVGSVVSRLNLNEPEIKQACFYQRVHDKCTQLQELRWSHVELHDDTEMLVSIL
LPLPDLKKFELTSCSFTPTGIDCLITGLQRCQAIEELNLGHMKLGDAAIPKLVFGLCEMPSLKRLILNH
NSIGDDGCSRLAEALSSMHCMEEINLGHNKIGDLGLINIAAVLLEMQNLKRIDLSGNCPSPAGGEKLME
ALANCKHLEELILSRNDFGDGTAVKLALCLPHMNRLKILHLQHNNIGPAGGTELARALMACELLEEISL
SENNLGEGGIRALSEGLPRFEHLRKIELRLCGITDDASKLLSRGFQQCPAVEEIILSWNALGDGGAQVL
ASILPGMETLKMLDLEKNLIGACGATRLAEELVRCPEIQFIRLWDNPVPKGLAESLTSQDPRLCFSFC

>NLRP3-chicken
MAGEESTIFLEALEGLTLEDFQEFKKKLPHTDIKGGWNIGRDELEKVTHPSSLISYMGDSYGEGAAMDI
AISLFEEMNQRDLAEKILDEKVKEYKQKYTEHVAREFLQYKEANSCLGENLSVRDRYTNLTIARKSWDQ
HGDEPGDVSSDTVTTQTLLEPSKDGQVPPITVLVGASGMGKTMTIRKVMMEWVEGTLCTQFDYVFCIDC
KELSFSKEVSMVDLISKCCPQQRMPAGRILGNPEKILFIFDSFEALGLPLAQPKDELSTDPTEAKPLET
TLLSLLRRTVLPESSVLIATRPAALQSLGQCLEGKHYVEILGFSPAAREEYFHRYFGNDNKADVAFRFT
RGNEVLYSLCVIPVMSWTVCTVLERELYERNQLLACSKTTTQMIMFYLSWLMKHRVSNAWQNLQQFLHK
LCSLAADGIWKHKVLFEEKEIEDQGLNQPQLLSLFLNEKGLEKGTDHVNVYSFSHLHLQELFAAMFYVL
EDQDGMVSDSRILAKDVNMLLESYHTSRMDLNVTVRLLFGLVNPKSVEYAGEGIGCRISLQPQEDLLRW
LQTRPRGTSHPREVMKIEDLDTFHLLFETNEKSFVQSVLGSFTGIALQDVKLTLYDQAALCFCIKQWAG
LLSVTLWSCSFHQQHHRQEPAKGLPRQSWRQEELHSPLHPLCQALGHPGSSLQSLRLQWCGLTEGDSGA
LGTLLATLPSLVHLELGDGALGDDGVRMLCAGLRQPGCQLRVLR

>NLRC3-chicken
MEEAWISRYRRQLMRSISPQFLEEIIGYLQRLDLLTVEEAGRAQEASSLPEQVRAVVDILAGKGSHASQ
TLQSFIETTNSQLYPLCAEPMVQKHLESLQSFYGNSLEIGSLQRLTNLLLVEGLTDIQQKEHDILQMEM
TKGLRNVSKSIPLEKLFLPLSKVSIPPRISVTMGVAGIGKSTLVKLFVGRWAKGLINRDIMFVLPLTFR
ELNTYEKLSAERLIRSSFPHITEPNYISAGAARTLLILDGLDEFKTPLDFSNTVACTDPKKEIQVDNLI
TNIIRGNLLQEASIWVTSRPTAASQIPGGLVDRMTEIRGFRATEMKDFLDQMFLDNKDLSGQVLHHIKA
NRSLHIMCTVPGFCRIYGSSIGYYLKNSTGQPQEMTVAPKTLSEIYSYYFKMALSSDWPERRRETLRIE
QAVNNSKKVMGSLGRLAFYGLLKRKHVFYEQDMKAYGIDLSLLHSSLCSRLLLKEDMQPTTAYYFSHLT
IQEFLAAIYYYTAAKRAIFDLFTESGMSWPKLGFLNHFKSAVQRSLQAEDGQLDIFVRFLSGLLSPQVN
QLLSGWLLVKDEHNSYRSQAISFLQGCLNTNYVISSQTVNTVHCLHEIQHTEIAKAVEEAMKNESLAGM
LTPVNCSALAYLLQVSDVCVEETNLSNCLTYNICKSLLPPLLFCHSLRLDNNQFKDNVMELLGSMLSVK
DCQIQRLSLAENQICNKGAKALARSLMVNRSLTVLDPRSNSIGPSGAKALADALKKNQVLLSLRLQHNV
IKEEGAAALAEALLTNRRLITLHLQKNSIGAHGARKLAEALAQNCSLKELMLSSNSVGDNGSVALAEAL
KVNHSLQSLDLQSNFISNTGVSALTAALCSNKGLTDLNLRENSISKEGGPAIAHALRTNCTLRKLDLAA
NLLHDEGGKAIALAMKENRALTSLHLQWNFIQTQAAVALAQALQSNDSLASLDLQENAIGDEGMAALAA
ALKVNTTLADLHLQVASISAAGAQVLAEALMVNNSLQVLDLRGNSISVAGAKAMANALKVNRSLRWLNL
QENSLGMDGAICIATALRGNHGLTYVNLQGNRIGQSGAKMISDAIRTNSPDCVVVV

>NOD1-avian-ancestor
MEGQLCANLEISVEKPPGASPPSFIALLKVYRELLVSRIRNTQCLIDNLIKNDYFSTEDAEIVVQFPTQ
ADKVRKILDLVQSKGEEVSEYFICVLQKVTDAYYELQPWLDEIGYEPSENICSKPVVNTDPVSRYCQKL
RYELGRDSKFVMSYAQREEMLLEEIYSNSIMELVSFTNESLGEVCQLEALFDDAVGLINEDGETIYVFG
DAGIGKSILLQKIQSLWARKELDIGAKFFFRFRCRMFSCFKEDEAICLKDLLFKYNCYPDQDPTEVFHH
ILQFPHTVLFTFDGFDEIYSNFDLSSVPEMCSPNEPIHPLVLLVSLLRGKLLKGSKKILTARTGTEIQR
NIIRKKVLLRGFSSNNLKEYTAMFFKDEGQRTLVLNQLEANPNLCSLCSVPLFCWIIFKCYEHFHSMFD
SHELPDCSVTLTDVFLLMIEVHLNRSLKTSLLKNNTRSQAEMFKSRKETLLALGKMAYKGMENSFFIFE
QEEVSSANISEGDLQLGFLRTVKGYSRCDNQSTYEFLHLTLQSFFTALFLVIEEKVGAKELLQFFNECS
STETAQPTCLRIPWLKKQLAGEDPFRNNEHFHFTNLFLCGLLSRSKQKLFRHLVSPAVIKRKRKTLITY
LGESMKSHLKGLTRSRLPNYNQVQVQPNFVWMLRCLYETQSEKVGKLAAKRMHANYIKLTYCNAYSADC
SAISFVVHHFQKRLALDLDNNNINDYGVKQLLPCFSKLAVIRLSVNQVTDHGVRILYEELSKYQIVSFL
GLYNNQITDVGAKYVAKLIEECSSLEYVKIGANKITSEGGKCLAQAIQKSKTMFEIGMWGNQVGDEGAK
AFAEALRNHPRLTNVSLAFNGITTEGGKSIAEALQHNNSVKIFWLTKNELDDEAAMSFAEMLKVNKKLV
HLWLIQNQITAKGVKYLSEALKENTAIKEVCLNGNLISQEEAKAFENEERIICF

>NOD1-mammalian-ancestor
MEEQGHSEMEIIPSESHSHIKLLKINRELLVTHIRNTQCLVDNLLKNDYFSAEDAEIVCACPTQPDKVR
KILDLVQSKGEEVSEFFLYVLQQLADAYVDLRPWLSEIGFSPSQLIQSKAVVNTDPVSRYTQQLRHQLG
RDSKFMLCYAQKEELLLEEIYMDTIMELVGFSNESLGSLGSLACLLDHTTGILNEQGETIFVFGDAGVG
KSMLLQRLQSLWATGRLDAGVKFFFHFRCRMFSCFKESDTLCLQDLLFKHYCYPEQDPEEVFAFLLRFP
HTALFTFDGLDELHSDFDLSRVPDSSCPWEPAHPLVLLANLLSGKLLKGASKLLTARTGIEVPRQLLRK
KVLLRGFSPSHLRAYARRMFPERAVQERLLDQLEANPNLCSLCAVPLFCWIIFRCFQHFHAAFEGSPQL
PDCTVTLTDVFLLVTEVHLNRTQPSSLVQRNTRSPAETLRAGRDTLRSLGQVAHRGMEKSLFVFSQEEV
QASELQEGDLQLGFLRALPELGPGGDQQSYEFFHLTLQAFFTAFFLVVDDKVGTRELLRFFQEWTPPGE
AAAASCYPPFLPFQCLGGSGLAGEDPFKNKDHFQFTNLFLCGLLSKAKQKLLRHLVPAAALRRKRKALW
AHLFASLRSYLKSLPRVQSEGFNQVQAMPTFIWMLRCIYETQSQKVGQLAARGICANYLKLTYCNACSA
DCSALSFVLHHFRKRLALDLDNNNLNDYGVRELQPCFSRLTVIRLSVNQITDSGVKVLCEELTKYKIVT
FLGLYNNQITDVGARYVAKILDECKGLTHLKLGKNKITSEGGKCLALAVKNSKSIFEVGMWGNQIGDEG
AKAFAEALRNHPSLTTLSLAFNGISTEGGKSLAWALQQNTSLRIFWLTKNELDDEVAESLAEMLKVNQT
LKHLWLIQNQITAKGTAQLADALQKNTGITEICLNGNLIKPEEAKVFEDEKRIICF

>NLRC5-avian-ancestor
MQPPFQNDDLDLEAAVASVRPQLVEFLSHRPDWLLTTSQRFLPGVVLNGLDGITDHREKVSVLLDLLEK
AGPATWKQFAQYLCMECDLPLDLEILLMSSAGEGNFTQKQEACTDVSARSSVPKGFARRRRRSSSVSDK
DAKQQRLDSAEKYRHLLIDSICQRYGSRRAAGAAAQEPTQPLAFNQAFVNLVIRQSKASRLKERTDKPR
EDVAGAPEPEECVDTAVKVSDLFGSVVPSGTTKVIFLFGKPGTGKTMLMHRICQKWAEGVLHQFLFTFL
FEFRQLNLLKRKLTLKELLFDLFLQPEDSPDAVFQYLLENAQHTLIIFDGLDEFVGNMDVSSSSKGGPT
LSSRMSISELFADLCHGNLLPGCTVLVTSRPKRLPDFLLNTVDLLAEIWGFDHEKVEEYVSHYFHHHSF
KEQAIAHLKNNTKLLSMCLIPALCYVVCICLEYLLLKHQMSVELPQTMTQFYIKMLLIFINKQQGERAG
DEETQLNCNKKAILGLCDLALKGLEAKKLVFYVGDIPEHVKEFASLHGLLTVFEVKTSGTCPEAGYAFV
HLSLQEFFAALCLMVSKRVDKNYLKKKFSLKSKWTLKNEAKTEFMESFHIFLSGLSSKECRTFLMLLAE
QDEAWVQDKQDAILQSLKKLAATQLTGPKVIELCHCTFETQDLEVAQHIGSLLNFKYEFKNFRLTPLDM

SALVFVINSGQDLTHLDFAGCLLDIDCLEVLAGCKNVEHLSFRSRRCGDDFAAALSKGLRGMGSLKKLE
LTGGSITAEGMTNLVQALSHCLQLEEINLQDNRIRDPEVKRVMELFSRMEKLKKIDLSNNNLSLNAVLI
LAKEVIACQNATELRVRKDTVIISFSGPSGKVPRSLDLRWEENKEHVIPTRHLKLCLQDRCLSSQHAKE
IVAILQSCPCLSEVDLSGNKLGDEGCSFLLESLPWISISKQLDLSHNRLSVTGICSLLKAVNTCQKVME
VQVSLCHKTAVLRFTEDREFASLPPREEPVFSTDDQQYGEENQTPTASKKIRLTYCRFQASDLEKLCAV
LKECGSISELDLSNNYLGDEGLLQLFQFLPNLKMLRSLKLNDNQLSLNSVFFLAQSLSTLEHIKTMNLS
LGHMQVVHLTFGERIRVRSTSRWRSDSFLAHPKHVANGRCFRLRDCRVGPEDVTRLCQILAQCPQLTEI
DLSGNSLSDQSIERLLSFLPYLCQLTLLSIRNNTFSPRCTVLLINSINLCERIRMVEVRSSENAFLHLR
ASTQSQKTSCRLTDCAIGQGQIEELCRVLEQHGCLAELDLSRNQLGDEGLRCLLDHLHRVPVTCSLNLS
HNRISQDGVLHLINAFATSGNTTEVQVSLCSKATLIIKLTSRDDPRKILRLAECNFQPEHLEKLCLLLE
KCTDLTEYISSNNNVTVQAAERLLHSLRKTLGPLKISIEEPWVCKESVTNLLELAVQACGNITEITICK
DKNLFQLGVRFPHCLEKVESVVSRLNLYKPEIKHACFYQRVREKCAQLQELRWSHVELHNDEAEMLVSI
LLPLPELKKFGLTSSSIMPTGIDYLITGLQNCQAIEELNLGHMKLSSAAIPKLVLGLCEMPSLKRLILN
HNSIGNDGCSRLAEALRNMHYMEEINLSHNKIGDPGLINLAAVLLEMQNLKRIDLSGNCPSPAGGEKLM
EALAYCKHIEELILSRNGFGDRTAVKLALCLPHMTNLKILHLQHNNIGPAGGMELARALVVCELLEEIS
LSENDLGEGSIHALSEGLPCFEHLRKIDLKLCGITDDASKSLSHGFRQCPAMEEIILSWNALGDGGAQE
LASVLPGMEKLKMLDLEKNRIGACGATKLAEELAKCPEIQFIRLWDNPVPKGLAESLTSQDPRLCFSFC

>NLRC5-mammalian-ancestor
MDPVSLQLGNKNLWSWLVRLLSKDPEWLNAKVKFFLPNMDLDSRNEALDPTQRVILQLNKLHAQGPATW
QSFIHCVCMELEVPLDLEVLLLSTWGHEDGFPSQLGAGEESQPESQLHHGLKRPHQSCGSSPRRKQCRK
QQLELAKKYLQLLRTSAQQRYGSRIPGPGQPLAFHQAYVPPILQWSRASAPLDTQEGAIMGDPKAEDGT
DVSILDLFNTKANKGPRVTVLLGKAGMGKTTLAHRLCQKWADGQLDRFQALFLFEFRQLNLITRFLTLP
QLLFDLYLSPEADPDAVFQYLEENADQVLLIFDGLDEALQPCSSKETVGPDDPGSALTLFSGLCHGTLL
PGCWVMATSRPGKLPACLPTEAAMVHMWGFDGPRVEEYVNHFFSDQPSQEAALAELRANGRLRSMCAVP
ALCQVACLCLHYHLLPGSSPGQSAALLPTMTQLYVQMVLALSPRGHLPATSLLGLGEVALRGLETGKVI
FSAEDIPPPLMAFGAAHSLLTSFCIRTGPGHQETGYAFTHLSLQEFFAALHLMASPKVDKDTLTHHVTL
NSRWVLRTKARLGLSDHLPTFLAGLASRTCRPFLSHLAQRDEVWVGAKQAAVVQVLRKLATRKLTGPKV
VELCHCVGETQEPELASLIAQSLPYQLPFHNFPLTCADLATLTNILEHRDAPIHLDFDGCPLEPHCPEA
LVGCGQVENLSFKSRKCGDAFAEALSRSLPTMGSLKMLGLAGSKITARGISHLVQALPLCPQLEEVSFQ
DNQLKDQEVLNIVEVLPCLPRLQKLDLSRNSVCMSTLLCLAKVAVTCPTVRMLQVREADLIFLLSPPTE
TAAELQGAPDLQGNDSQRKGAQSRSLTLRLQKCQLRVHDAEALIALLQEGPHLEEVDLSGNQLEDEGCR
LMAEAASQLHIARKLDLSDNGLSVAGVHCVLRAVSTCRTLAELHISLLHKTVVLTFAQEPEEQEGIQKR
AAFLDSLMLQMPSELPLSSRRIRLTHCGFQAKHLEQLCKALGGSCHLGHLDLSGNALGDEGVAQLAQLL
PGLGPLQSLNLSENGLSLDAVFSLAQCFSTLQWLFHLEISFESQHILLRGDRTGRDMLAAGSLPEFPAG
AQFLGFGQRCIPRSFCLRECQLEPLSLTHLCDTLEKCPGPLEVQLSCEFLSDQSLETLLHRLPQLPQLS
LLQLSQTGLSPRSPFLLADIFSLCPRVQKVDLRSLRHATLHFRSNEEQEGVCCRFTGCSLSQEHVEPLC
WLLSKCKDLSQLDLSANLLGDSGLRCLLECLPQVPISGSLDLSHNSISQESALYLVETLPSCPRVREAS
VNLGSEQSFRIHFSREEEAGKTLRLSECSFRPEHVSRLATGLSQSLQLTELTLTQCCLGLEQLTILLSL
VRRPAGLFSLRVEEPWVGRAGVLTLLEVCAQASGSVTEISISETQQQLCVQLEFPRQEENPEAVALRLA
HCDLGTHHSLLVRQLMETCARLQQLSLSQVNLCEDDDASSLLLQSLLLSLSELKTFRLTSSCVSTEGLA
HLASGLGHCHHLEELDLSNNQFGEEGTKVLMGALEGKCRLKRLDLSHLPLDSSTLALLTQGLSHMTLLQ
SLSLSRNSIGDVGCCHLSEALRAATSLEELGLSHNQIGDTGAQHLAAILPGLPELRKIDLSANSIGPAG
GVQLAESLALCRHLEELMLGCNALGDPTALGLAQGLPQHLRVLHLPSSHLGPEGALSLAQALDGCPHVE

EISLAENNLAGGVPHFCKGLPLLRQIDLVSCKIDDQTAKPLAASFTLCPALEEILLSWNLLGDEAAAEL
AQVLPQMGRLKRVDLEKNQITACGAWLLAEGLAQGSGIQVIRLWNNPIPPDMAQRLQSQEPRLDFAFFD
NQPQAP
