# Supplementary material for: Structural and Evolutionary Adaptation of NOD-Like Receptors in Birds
Source: Biomed Res Int. 2021 Apr 29;2021:5546170. doi: 10.1155/2021/5546170 (PMC8105094; doi:10.1155/2021/5546170)
Supplement: Supplementary Materials — S1: RIP2 alignment. S2: primers and chicken sequences and ancestral sequences. S3: avian sequences accession number. Supplementary Table 1: sites found to be under positive selection. [file 5546170.f1.zip › S3 avian sequences accession number.pdf]

	NOD1 (47)	NLRP (38)	NLRC3 (42)	NLRC5(32)	RIP2(38)
1	NOD1-chicken	NLRP3-chicken	NP_849172.2 NM_178844.3 Homo sapiens	AEY11256.1 JQ044414.1 Gallus gallus	NP_003812.1 NM_003821.6 Homo sapiens
2	NP_006083.1 NM_006092.4 Homo sapiens	AAI43360.1 BC143359 Homo sapiens	NLRC3-chicken	NP_115582.4 NM_032206.4 Homo sapiens	RIP2-chicken
3	XP_011594380.1 XM_011596078.1 Aquila chrysaetos canadensis	XP_010709125.1 XM_010710823.1 Meleagris gallopavo	XP_010717657.1 XM_010719355.2 Meleagris gallopavo	XP_009641653.1 XM_009643358.1 Egretta garzetta	XP_015711414.1 XM_015855928.1 Coturnix japonica
4	XP_003207269.1 XM_003207221.2 Meleagris gallopavo	KFV98054.1 KK587607.1 Fulmarus glacialis	XP_009276821.1 XM_009278546.1 Aptenodytes forsteri	KFM02684.1 KL225750.1 Aptenodytes forsteri	KFO74077.1 KL447477.1 Cuculus canorus
5	XP_010560686.1 XM_010562384.1 Haliaeetus leucocephalus	KFP20708.1 KK502100.1 Egretta garzetta	XP_010565343.1 XM_010567041.1 Haliaeetus leucocephalus	KFV17315.1 KL249984.1 Pterocles gutturalis	XP_005150905.1 XM_005150848.2 Melopsittacus undulatus
6	XP_010300536.1 XM_010302234.1 Balearica regulorum gibbericeps	XP_009487832.1 XM_009489557.1 Pelecanus crispus	XP_005436874.1 XM_005436817.2 Falco cherrug	XP_010153118.1 XM_010154816.1 Eurypyga helias	XP_010181516.1 XM_010183214.1 Mesitornis unicolor
7	XP_010294769.1 XM_010296467.1 Phaethon lepturus	KFM10527.1 KL226261.1 Aptenodytes forsteri	XP_009880270.1 XM_009881968.1 Charadrius vociferus	XP_009892792.1 XM_009894490.1 Charadrius vociferus	XP_014820946.1 XM_014965460.1 Calidris pugnax

8	XP_010205023.1 XM_010206721.1 Colius striatus	KFQ68021.1 JJRF01090565.1 Phaethon lepturus	XP_011598028.1 XM_011599726.1 Aquila chrysaetos canadensis	XP_009983127.1 XM_009984825.1 Tauraco erythrolophus	XP_014726238.1 XM_014870752.1 Sturnus vulgaris
9	XP_010190983.1 XM_010192681.1 Mesitornis unicolor	KFV12353.1 KL241692.1 Pterocles gutturalis	XP_005244217.1 XM_005244160.1 Falco peregrinus	XP_010006813.1 XM_010008511.1 Chaetura pelagica	XP_017681969.1 XM_017826480.1 Lepidothrix coronata
10	XP_010075327.1 XM_010077025.1 Pterocles gutturalis	KGL99219.1 KL873572.1 Charadrius vociferus	XP_009478793.1 XM_009480518.1 Pelecanus crispus	EMC79680.1 KB375933.1 Columba livia	KQK75542.1 LMAW01002926.1 Amazona aestiva
11	XP_010146090.1 XM_010147788.1 Eurypyga helias	XP_008934994.1 XM_008936746.1 Merops nubicus	XP_005150824.1 XM_005150767.1 Melopsittacus undulatus	XP_014141836.1 XM_014286361.2 Falco cherrug	XP_005042333.1 XM_005042276.2 Ficedula albicollis
12	XP_010136979.1 XM_010138677.1 Buceros rhinoceros silvestris	KFP94830.1 JJRL01079955.1 Haliaeetus albicilla	KFP67380.1 KK523803.1 Cariama cristata	XP_013152548.1 XM_013297094.1 Falco peregrinus	XP_005479583.1 XM_005479526.2 Zonotrichia albicollis
13	XP_009970885.1 XM_009972583.1 Tyto alba	KFQ33932.1 KK808678.1 Mesitornis unicolor	KFV80758.1 KL206272.1 Struthio camelus australis	KFP82096.1 KL373453.1 Apaloderma vittatum	XP_005517100.1 XM_005517043.2 Pseudopodoces humilis
14	XP_009987988.1 XM_009989686.1 Tauraco erythrolophus	KFV56684.1 KK394856.1 Tyto alba	KFQ12821.1 KK681585.1 Leptosomus discolor	KFO71370.1 KL447260.1 Cuculus canorus	KFP59105.1 KL334407.1 Cathartes aura

15	XP_010005792.1 XM_010007490.1 Chaetura pelagica	KFQ43372.1 JJRH01073841.1 Nestor notabilis	EMC86605.1 KB375484.1 Columba livia	KFW81197.1 KL671099.1 Manacus vitellinus	EOA94323.1 KB744834.1 Anas platyrhynchos
16	XP_010019083.1 XM_010020781.1 Nestor notabilis	KFO97775.1 KL217671.1 Calypte anna	KFW03889.1 KK565216.1 Eurypyga helias	XP_012425009.1 XM_012569555.1 Taeniopygia guttata	KFP60755.1 KK504190.1 Cariama cristata
17	XP_009946267.1 XM_009947965.1 Leptosomus discolor	KFO06954.1 KL477839.1 Balearica regulorum gibbericeps	KFQ93251.1 KL410017.1 Nipponia nippon	XP_008637076.1 XM_008638854.1 Corvus brachyrhynchos	KFQ90254.1 KK436006.1 Phoenicopterus ruber ruber
18	XP_009936361.1 XM_009938059.1 Opisthocomus hoazin	KFQ14462.1 KK685703.1 Leptosomus discolor	XP_010390880.1 XM_010392578.1 Corvus cornix cornix	XP_010401762.1 XM_010403460.2 Corvus cornix cornix	KFQ63971.1 KK492683.1 Pelecanus crispus
19	XP_009906304.1 XM_009908002.1 Picoides pubescens	KFR07514.1 KL411427.1 Nipponia nippon	KFQ47237.1 KK936178.1 Nestor notabilis	XP_014110900.1 XM_014255425.1 Pseudopodoces humilis	KFM04929.1 KL225928.1 Aptenodytes forsteri
20	XP_009877889.1 XM_009879587.1 Charadrius vociferus	KFP59004.1 JJRQ01066678.1 Cariama cristata	KFQ30606.1 KK803699.1 Mesitornis unicolor	XP_014124554.1 XM_014269079.2 Zonotrichia albicollis	KFQ74688.1 KK452971.1 Phaethon lepturus
21	XP_009871122.1 XM_009872820.1 Apaloderma vittatum	KFV14857.1 KL463662.1 Tauraco erythrolophus	XP_009584531.1 XM_009586236.1 Fulmarus glacialis	XP_009089123.1 XM_009090875.1 Serinus canaria	KFQ07057.1 KK659212.1 Haliaeetus albicilla

22	XP_009704884.1<br>XM_009706582.1<br>Cariama cristata	XP_012984385.1<br>XM_013128931.1<br>Melopsittacus undulatus	KFO13787.1<br>KL498635.1<br>Balearica regulorum gibbericeps	KFV79923.1<br>KL206199.1<br>Struthio camelus australis	KFW05819.1<br>KK568428.1<br>Eurypyga helias
23	XP_009812730.1<br>XM_009814428.1<br>Gavia stellata	EMC82280.1<br>KB375693.1<br>Columba livia	KFR16430.1<br>KK735903.1<br>Opisthocomus hoazin	EOB01028.1<br>KB743139.1<br>Anas platyrhynchos	KFW67401.1<br>KL225093.1<br>Pygoscelis adeliae
24	XP_009638465.1<br>XM_009640170.1<br>Egretta garzetta	KFP73142.1<br>KK826479.1<br>Acanthisitta chloris	KFP12002.1<br>KK501007.1<br>Egretta garzetta	KFW60959.1<br>KL224535.1<br>Pygoscelis adeliae	KFU96217.1<br>KN127212.1<br>Chaetura pelagica
25	XP_009686170.1<br>XM_009687875.1<br>Struthio camelus australis	XP_008924714.1<br>XM_008926466.1<br>Manacus vitellinus	KFP76274.1<br>KK830666.1<br>Acanthisitta chloris	XP_013032006.1<br>XM_013176552.1<br>Anser cygnoides domesticus	KFR17723.1<br>KK736152.1<br>Opisthocomus hoazin
26	XP_009581032.1<br>XM_009582737.1<br>Fulmarus glacialis	XP_012429272.1<br>XM_012573818.1<br>Taeniopygia guttata	KFP91701.1<br>KL391228.1<br>Apaloderma vittatum	XP_011572067.1<br>XM_011573765.1<br>Aquila chrysaetos canadensis	KFR01688.1<br>KL410965.1<br>Nipponia nippon
27	XP_009570032.1<br>XM_009571737.1<br>Cuculus canorus	XP_005426581.1<br>XM_005426524.1<br>Geospiza fortis	KFW75266.1<br>KL669029.1<br>Manacus vitellinus	XP_010581551.1<br>XM_010583249.1<br>Haliaeetus leucocephalus	KFP01549.1<br>KL218031.1<br>Calypte anna
28	XP_009469903.1<br>XM_009471628.1<br>Nipponia nippon	XP_010390336.1<br>XM_010392034.1<br>Corvus cornix cornix	KFQ69060.1<br>KK437097.1<br>Phaethon lepturus	XP_009931924.1<br>XM_009933622.1<br>Opisthocomus hoazin	KFP88042.1<br>KK846543.1<br>Acanthisitta chloris
29	XP_009485014.1<br>XM_009486739.1<br>Pelecanus crispus	KFV79209.1<br>KL206137.1<br>Struthio camelus australis	XP_005523203.1<br>XM_005523146.1<br>Pseudopodoces humilis	KFP40615.1<br>KK747278.1<br>Chlamydotis macqueenii	KFO87475.1<br>KL514241.1<br>Buceros rhinoceros silvestris

30	XP_009281840.1 XM_009283565.1 Aptenodytes forsteri	KFV66995.1 KL215850.1 Picoides pubescens	KFU86376.1 KN126074.1 Chaetura pelagica	XP_009289022.1 XM_009290747.2 Aptenodytes forsteri	KFP33539.1 KK550202.1 Colius striatus
31	XP_009084409.1 XM_009086161.1 Serinus canaria	KFP48228.1 JMFT01165428.1 Cathartes aura	KFP33880.1 KK551428.1 Colius striatus	XP_012955436.1 XM_013099982.2 Anas platyrhynchos	KFO57232.1 KK718509.1 Corvus brachyrhynchos
32	XP_009080014.1 XM_009081766.1 Acanthisitta chloris	KFQ86160.1 KK419318.1 Phoenicopterus ruber ruber	KFO88859.1 KL518566.1 Buceros rhinoceros silvestris	XP_013804078.1 XM_013948624.1 Apteryx australis mantelli	XP_010403308.1 XM_010405006.1 Corvus cornix cornix
33	XP_008946501.1 XM_008948253.1 Merops nubicus	KFP43339.1 KK755995.1 Chlamydotis macqueenii	KFV66077.1 KL215669.1 Picoides pubescens		KFO13799.1 KL498661.1 Balearica regulorum gibbericeps
34	XP_008931768.1 XM_008933520.1 Manacus vitellinus	XP_005030014.1 XM_005029957.4 Anas platyrhynchos	KFO63309.1 KK719436.1 Corvus brachyrhynchos		KFV81187.1 KL206309.1 Struthio camelus australis
35	XP_008632142.1 XM_008633920.1 Corvus brachyrhynchos	XP_013053803.1 XM_013198349.1 Anser cygnoides domesticus	XP_009813859.1 XM_009815557.1 Gavia stellata		KGL77642.1 KL890349.1 Tinamus guttatus
36	XP_008502603.1 XM_008504381.1 Calypte anna	XP_013800818.1 XM_013945364.1 Apteryx australis mantelli	XP_005054364.1 XM_005054307.1 Ficedula albicollis		KFZ59450.1 KL260561.1 Podiceps cristatus
37	XP_005510067.1 XM_005510010.1 Columba livia	KFZ66821.1 KL277519.1 Podiceps cristatus	XP_012432118.1 XM_012576664.1 Taeniopygia guttata		KFQ47898.1 KK937278.1 Nestor notabilis

38	XP_005519048.1<br>XM_005518991.<br>1<br>Pseudopodoces<br>humilis	XP_010166178.1<br>XM_010167876.<br>1<br>Antrostomus<br>carolinensis	XP_005425321.1<br>XM_005425264.2<br>Geospiza fortis		KFV95703.1<br>JJRN01099669<br>.1<br>Fulmarus<br>glacialis
39	XP_005424013.1<br>XM_005423956.<br>1<br>Geospiza fortis		KFV16485.1<br>KL465932.1<br>Tauraco<br>erythrolophus		
40	XP_005481045.1<br>XM_005480988.<br>1<br>Zonotrichia<br>albicollis		KFO71219.1<br>KL447254.1<br>Cuculus canorus		
41	XP_005445923.1<br>XM_005445866.<br>1<br>Falco cherrug		XP_010077294.1<br>XM_010078992.1<br>Pterocles<br>gutturalis		
42	XP_005242310.1<br>XM_005242253.<br>1<br>Falco peregrinus		XP_009090413.1<br>XM_009092165.1<br>Serinus canaria		
43	XP_005143803.1<br>XM_005143746.<br>1Melopsittacus<br>undulatus				
44	XP_005040913.1<br>XM_005040856.<br>1<br>Ficedula<br>albicollis				
45	XP_002196320.1<br>XM_002196284.<br>2<br>Taeniopygia<br>guttata				

46	AFS49704.1<br>JX465487.1<br>Gallus gallus				
47	AFP44845.1<br>JQ868803.1<br>Anas platyrhynchos				
