# Supplementary material for: Structural and Evolutionary Adaptation of NOD-Like Receptors in Birds
Source: Biomed Res Int. 2021 Apr 29;2021:5546170. doi: 10.1155/2021/5546170 (PMC8105094; doi:10.1155/2021/5546170)
Supplement: Supplementary Materials — S1: RIP2 alignment. S2: primers and chicken sequences and ancestral sequences. S3: avian sequences accession number. Supplementary Table 1: sites found to be under positive selection. [file 5546170.f1.zip › Table 1_revised.docx]

| **Gene** | **NOD1** | **NLRC3** | **NLRC5** | **NLRP3** | **RIP2** |
| --- | --- | --- | --- | --- | --- |
| **No. of**  **Species** | 47 | 42 | 32 | 38 | 38 |
| **lnL M7** | -26407.867678 | -21384.908770 | -42683.719049 | -20607.891898 | -8681.948583 |
| **lnL M8** | -26366.328328 | -21357.222901 | -42528.551497 | -20584.141639 | -8667.885388 |
| **-2lnΔL** | 83.08 | 55.37 | 310.34 | 47.50 | 28.13 |
| **PAML M8** | 9, 14, 93, 115, 181, 490, 574, 639, 640, | 145, 382, 724 | 39, 45, 48, 118, 130, 160, 231, 318, 343, 705, 715, 750, 820, 828, 880, 945, 1013, 1016, 1024, 1052, 1109, 1146, 1211, 1217, 1245, 1246, 1390, 1391, 1413, 1415, 1416, 1491, 1575 | 230, 410, 438, 593, 636 | 324, 352, 412, 440 |
| **SLAC** | 93(0.074), 115(0.036), 181(0.097), 476(0.080), 686(0.069), 697(0.017) | 2(0.005), 351(0.072), 480(0.091), 759(0.013) | 44(0.075), 66(0.046), 118(0.097), 159, 203(0.016), 224(0.060), 342(0.051), 343(0.011), 846(0.077), 852, 888(0.090), 896(0.053), 945(0.013), 995(0.064), 1013(0.054), 1050(0.095), 1139(0.086), 1144(0.059), 1152(0.053), 1211(0.022), 1217(0.015), 1395(0.020), 1396(0.070), 1416(0.083), 1446(0.090), 1491(0.070), 1575(0.078), 1617(0.054), 1663(0.027), 1800(0.052) | 136(0.021), 350, 356(0.076),  373(0.050), 544(0.070), 623,  655(0.032), 670, 679(0.080),  703(0.043), 727(0.049), 732(0.086) | 371(0.032),  412(0.007) |
| **FEL** | 7, 9(0.072), 13, 93(0.016), 115(0.004), 121, 140(0.053), 155, 175, 276, 343, 359(0.070), 448, 476(0.021), 513(0.073), 559(0.066), 633(0.043), 640(0.009), 678, 686(0.001), 697(0.012), 744, 925 | 2(0.001), 41, 73, 87(0.0003), 145(0.076), 228(0.062), 341(0.087), 351(0.063), 480(0.047), 759(0.012), 804, 1014 | 32, 53, 56, 66(0.007), 99, 123, 132,  159, 183, 203(0.003), 224(0.014), 242, 267, 342(0.009), 343(0.002), 494, 544, 575, 622, 716, 723, 750(0.040), 751, 754, 807,  828(0.050), 846(0.023), 852(0.003), 880(0.098), 888(0.039), 896(0.013),  945(0.010), 962, 964,  969, 973(0.050), 989(0.084),  995(0.037), 1002, 1013(0.076),  1024(0.045), 1041, 1050(0.036),  1100, 1103, 1122(0.072),  1139(0.029), 1141(0.091), 1144(0.010),  1152(0.018), 1211(0.006), 1217(0.0008),  1241, 1246(0.025), 1273,  1305, 1395(0.003), 1396(0.014),  1399, 1411(0.086), 1415(0.014), 1416(0.039), 1446(0.025), 1452(0.082), 1462, 1464, 1491(0.047), 1559, 1575(0.014), 1606, 1617(0.026), 1640, 1663(0.005), 1691, 1729(0.028), 1755, 1761, 1785, 1800(0.021) | 11(0.037), 60(0.056), 67, 108(0.065), 128(0.030), 136(0.022), 149(0.026), 235, 306(0.050), 356(0.013), 364(0.095), 373(0.041), 387(0.075), 390, 413(0.003), 423, 464(0.031), 544(0.016), 622, 633(0.082), 655(0.052), 703(0.026), 727(0.020) | 8, 12, 41, 222,  371(0.002), 409,  412(0.004), 464, 477 |
| **REL** | 14(105.3), 64, 93(243.2), 115(635.6), 140(444), 150, 166, 181(50.7), 271, 297, 313, 359(489.5), 456, 458, 476(101.6), 513(391.8), 559(387.0), 574(104.5),  578, 619, 633(509.7),  639(51.3), 640(812.4), 672, 686(2302.4), 697(1361.6) | 2(1.48296e+08), 87(3.91863e+07), 102, 145(54.3), 146, 228(101.4),  341(102.6), 351(152.1), 355, 362, 473, 480(239.6),  538, 568, 581, 686, 748, 749, 751, 759(139.8), 858, 888, 932, 976, 984 | 5, 30, 39(57.2), 40, 44(547.515), 48(51.22), 112, 113, 115, 118(184.8),  130  (82.5), 131, 135, 160(607.6), 336, 343(592.8),  349, 750(2056.1), 820(61.1), 828(402.7), 834, 843, 852(805.7), 861, 880(171.7),  888(66.1), 896(160.5), 945(2480.8),  973(120.5), 984, 989(424.3),  1013(1557.2), 1016(59.9), 1024(447.3),  1050(1484.0), 1052(102.4), 1111,  1117, 1120, 1122(268.5),  1139(892.1), 1141(91.5), 1144(335.3),  1146(56.8), 1211(2112.1), 1217(1721.4),  1228, 1245(163.2), 1246(1652.1),  1395(1333.8), 1396(198.2), 1398, 1411(57.9), 1412, 1413(181.3), 1415(408.7), 1416(1319.4), 1452(119.5), 1458, 1478, 1491(1455.7), 1535, 1575(3183.4), 1608, 1617(109.7), 1631, 1660, 1663(245.4), 1673, 1718, 1729(56.6), 1792 | 11(1336.1), 19, 33, 60(50.8),  88, 108(99.3), 112,  128(2837.3), 136(615.3), 149(3126.8),  161, 230(367.6), 306(525.7), 340, 356(1236.0), 364(50.7),  373(1674.1), 387(1335.0), 410(85.3),  413(2079.9), 438(369.0), 464(112.8),  544(74.3), 593(389.1), 633(1047.7),  641, 645, 647,  651, 655(107.1), 667,  672, 679(330.2), 703(547.6),  727(62.3), 732(262.6), 734 | 324(133.5), 384,  385, 386, 387, 388,  389, 390, 391,  392, 393, 394,  395, 397, 398,  399, 400, 401,  402, 404, 405,  406 |
| **Total**  **No. of**  **sites** | 16 | 8 | 51 | 24 | 3 |
